# A nested compartmental model to assess the efficacy of paratuberculosis control measures on U.S. dairy farms

**DOI:** 10.1371/journal.pone.0203190

**Published:** 2018-10-02

**Authors:** Malinee Konboon, Majid Bani-Yaghoub, Patrick O. Pithua, Noah Rhee, Sharif S. Aly

**Affiliations:** 1 Department of Mathematics and Statistics, University of Missouri-Kansas City, Kansas City, Missouri, United States of America; 2 Department of Veterinary Medicine and Surgery, College of Veterinary Medicine, University of Missouri, Columbia, Missouri, United States of America; 3 Department of Population Health Sciences, Virginia-Maryland College of Veterinary Medicine, Blacksburg, Virginia, United States of America; 4 Veterinary Medicine Teaching and Research Center, School of Veterinary Medicine, University of California Davis, Tulare, California, United States of America; 5 Department of Population Health and Reproduction, School of Veterinary Medicine, University of California, Davis, California, United States of America; University of Illinois, UNITED STATES

## Abstract

Paratuberculosis, also known as Johne's disease (JD), is a chronic contagious disease, caused by *Mycobacterium avium* subsp. *paratuberculosis* (MAP). The disease is incurable, fatal and causes economic losses estimated to exceed 200 million dollars to the U.S. dairy industry annually. Several preventive and control measures have been recommended; however, only a few of these measures have been validated empirically. Using a nested compartmental (NC) modeling approach, the main objective of this research was to identify the best combination of control and preventive measures that minimizes the prevalence and incidence of JD and the risk of MAP occurrence in a dairy herd. The NC model employs both MAP transmission estimates and data on pen movement of cattle on a dairy to quantify the effectiveness of control and preventive measures. To obtain reasonable ranges of parameter values for between-pen movements, the NC model was fitted to the movement data of four typical California dairy farms. Using the estimated ranges of the movement parameters and those of JD from previous research, the basic reproduction number was calculated to measure the risk of MAP occurrence in each pen environment as well as the entire dairy. Although the interventions evaluated by the NC model were shown to reduce the infection, no single measure alone was capable of eradicating the infection. The numerical simulations suggest that a combination of test and cull with more frequent manure removal is the most effective method in reducing incidence, prevalence and the risk of MAP occurrence. Other control measures such as limiting calf-adult cow contacts, raising calves in a disease-free herd or colostrum management were less effective.

## Introduction

Johne's disease (JD) is a chronic, infectious gastrointestinal disease of domestic and wild ruminants (i.e. cattle, sheep, goats, deer, and bison), caused by *Mycobacterium avium* subsp. *paratuberculosis* (MAP). Johne's disease is a global disease, which was first observed in dairy cows in 1895 [1]. Environmental viability studies found that MAP can survive for 8 months in feces at ambient conditions [2] and for 19 months in water at 38 degrees of centigrade. MAP remains viable in a desiccated state for up to 47 months [3]. MAP is difficult to eradicate from a herd, because the pathogen persists in the environment for a long time.

Upon infection with MAP, cattle undergo an asymptomatic period which can last for years. As the disease advances, an infected cow eventually becomes overtly symptomatic with decreased milk production, persistent diarrhea, and despite having no changes in appetite, the affected animal will exhibit progressive wasting and death if not culled. The very slow progression of JD and the difficulty in identifying infected animals due to imperfect diagnostic tests contribute to the difficulty in conducting MAP control measure studies. Accurate and detailed data from testing animals throughout their lifespan and at slaughter are often not available [4]. As a result, researchers have relied on mathematical and statistical models to study transmission dynamics of MAP. Infectious disease modeling and simulation can also be used to determine the effects of control policies and to identify the risk factors contributing to disease spread [5, 6, 7, 8].

Although between-pen cattle movement is an important factor in the spread of infectious diseases, it has been largely ignored in the modeling and analysis of various infectious diseases of farmed ruminants. Many studies [7, 8] include a diffusion term to capture the cattle movement, however, these models assume random movement of cattle throughout the farm rather than the purposeful movement of cattle between pens for management reasons. In addition, recent studies have used network data to study the dynamic of cattle trade movements [9]. However, most of network models only investigate the mobility patterns of individual animals among farms and ignore between-pen cattle movements within each farm. Without considering between- pen cattle movements it would be difficult to provide meaningful inferences pertaining to within and between-pen disease transmission dynamics.

The objective of this study was to create a nested compartmental (NC) model for MAP transmission which accounts for progression of the disease and also the movement of cattle between pens on a dairy. The model fitted to the cattle movement data and employed to assess control and preventive measures. Using the numerical simulations of the model we identified the most optimal intervention combinations yielding the greatest reduction of JD incidence and prevalence on a dairy.

## Materials and methods

### NC model justification and design

Cattle movements between pens can modify the contact rates between MAP in the environment, susceptible and infected cattle. The changes in cattle population and contact patterns are more complicated than concept of random mixing assumed in most infectious disease models. In practice, the continuous management-based changes in pen populations on a dairy can strongly influence MAP transmission dynamics and the effects of MAP control measures. Thus, an NC model for MAP transmission on a dairy farm was constructed by considering the states of MAP infection in dairy cattle as well as the frequency and patterns of cattle movements between pens on a dairy. The Cattle Movement (CM) model is a compartmental model formulated with a set of Ordinary Differential Equations (ODEs) and acts as the shell or outer layer of the NC model. The CM model represents different pens on a dairy and within each pen type, a compartmental MAP transmission model is embedded according to the age of cattle in the respective pen.

#### The cattle movement model

The CM model encompasses the different production stages common to large dairy herds (≥500 milking cows) which represented 47% of US dairies in 2007 [10]. The CM model was constructed by dividing the dairy into pen types that closely represent cattle housing on a dairy. The pens include the calf nursery, growing and breeding pens, post-calving (fresh), high and low milk production, and non-milking (dry) cow pens for a total of 14 pen types. Table 1 lists the pen types, descriptions and the expected residence times per given animal.

**Table 1:** Pen types, description and the approximate range of residence time on large (≥500 milking cows) US dairies.

Pen Number	Pen Type	Pen description	Residence time
1	Pre-weaned	The period between birth and weaning	2–4 months
2	Post-weaned	The period after weaning; the rumen is	6–12 months
		developed enough that the animal can	
		survive without milk	
3	Breeding	Breeding of female cattle (heifers) for	1–3 months
		their first pregnancy	
4	Pregnant	Pregnant cows	7–8 months
5	Springers	Heifers close to giving birth (calving)	6–8 weeks
6	Fresh milking	Cows in their first lactation that just	0–3 weeks
	or Hospital L = 1	calved (fresh) and started lactating	
7	Fresh milking	Cows in their second or greater	1–12 months
	or Hospital L>1	lactation	
8	High milking	First lactation cows in their high milk	1–12 months
	L = 1	production phase of their lactation	
9	Low milking	First lactation cows in their low milk	1–12 months
	L = 1	production phase of their lactation	
		(post-peak).	
10	High milking	Second or greater lactation cows in their	1–12 months
	L>1	high milk production phase of their	
		lactation	
11	Low milking	Second or greater lactation cows in their	1–12 months
	L>1	low milk production phase of their	
		lactation	
12	Dry cows	Cows that ceased milk production cows	4–8 weeks
		as in the case of late pregnancy	
13	Close up	Cows close to calving	1–7 days
14	Calving	The act of delivery or giving birth to	1–7 days
		a calf	

Note: There could be multiple pens of each type. The actual number of pens within a pen type can vary by herd size and dairy management.

**Fig 1:**
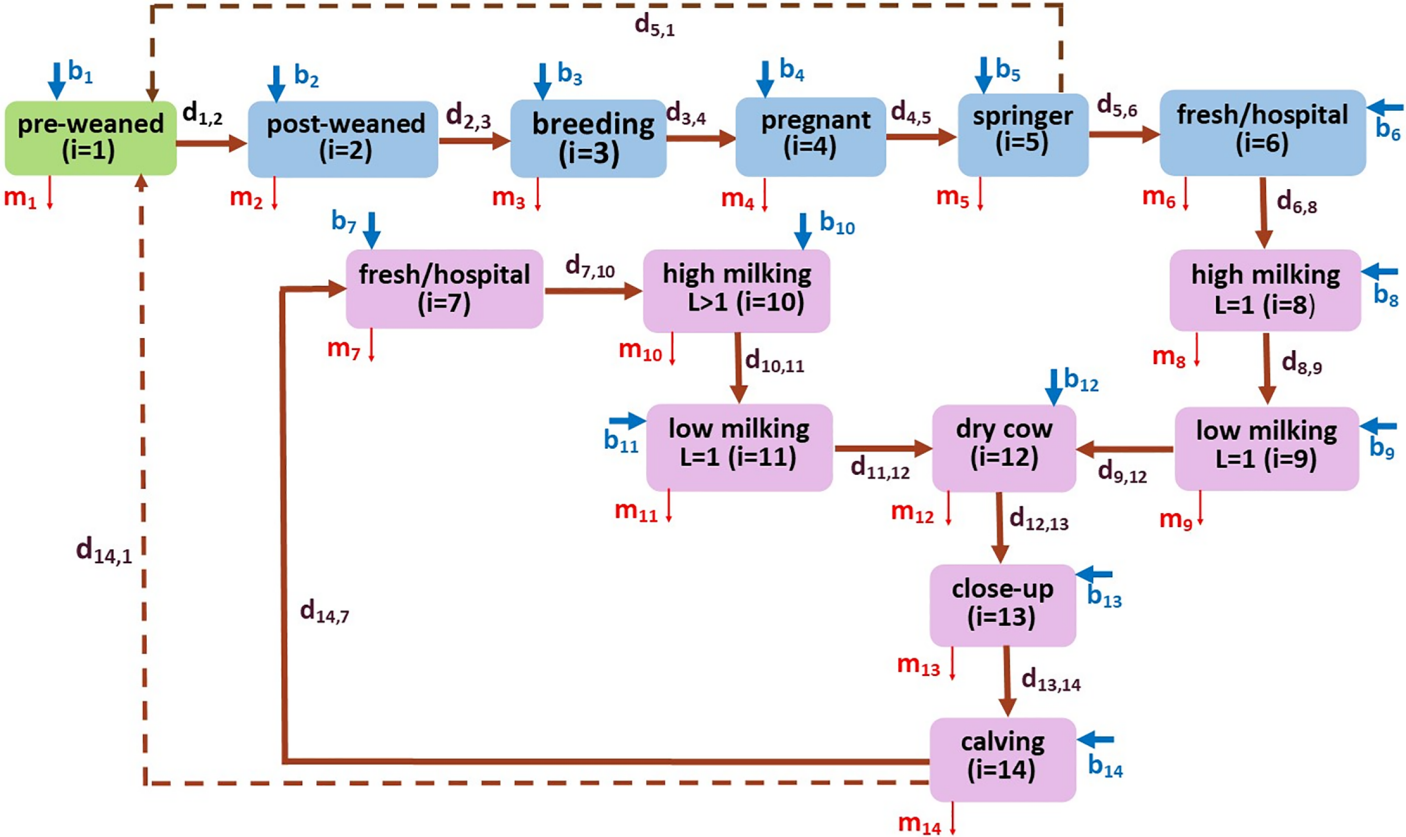
Compartmental diagram of cattle movements between different pen types (the actual number of pens can be higher, but the farm structure remains the same). Dashed lines represent the movement of newborn calves. The rate of moving from pen type *i* to pen type *j* is denoted by d_i, j_, for *i*, *j* = 1, …, 14. Birth and mortality rates are denoted by *b*_*i*_ and *m*_*i*_ for *i* = 1, …, 14, respectively.

Fig 1 is a flowchart of the CM model depicting the dynamics of moving dairy cattle between pens on a dairy. Although several dairy farms may have different pen structures, the illustrated flowchart is widely accepted among researchers and practitioners. Moreover, the flowchart represents the pen types, and there can be multiple pens of each type.

Calves born to nulliparous females also known as springers (female cattle which have never given birth before) in pen 5, are transported to the pre-weaned calf hutches, collectively identified here, pen 1. Also, calves born to parous females in pen 14, which are either uniparous (first lactation) or multiparous (second and greater lactation), are transported to pen 1. Once calves are weaned they are moved to pen 2, post-weaned group pens. On a dairy that raises female heifers for breeding, heifer calves are moved to pen 3 at breeding age and to pen 4 when pregnant. As the pregnant heifer approach calving (birth) they are moved to the springers pen, where they calve; or, depending on the dairy’s management, may be moved to an individual or group maternity pen. Because it is common for springers to calve in the close-up pens, the CM model was designed as such.

The recently calved females, now first lactation (uniparous) dams, are then moved to pen 6 after calving. Pen 6 (fresh/hospital) houses only recently calved first lactation cows separated from multiparous cows. This is a common management practice since older cows tend to be more dominant and may limit the younger smaller uniparous females’ access to feed. Furthermore, depending on the dairy’s management, pen 6 may also serve as a hospital pen to facilitate segregation of colostrum and milk produced in the first few days post-calving since both are not saleable for human consumption and hence are not milked into the bulk tank on the dairy.

Pen 8 houses high producing (high-milk production) cows. When milk production begins to decline, first lactation cows are moved to the low-production pen 9. Later in lactation and as milking cows approach calving, they will undergo dry-off, an industry term referring to the voluntary cessation of milking approximately 60 days prior to calving, a period necessary to replenish a cow’s body reserves and initiation of colostrum production in preparation for the new born calf. Dry-off cows are moved to pen 12 until before calving (usually 1 to 2 weeks) when they are then moved to the close-up pen (pen 13) and fed a different ration. Cows that start calving are moved to the calving (maternity) pen 14 and once calved they are moved to pen 7, the fresh/hospital pen.

Pen 7 houses multiparous (calved more than once) fresh cows (an industry term for female cattle that recently calved) to segregate and collect their colostrum and the first 1 to 7 days of milk, depending on the type of antibiotics used at dry-off to avoid antibiotic residues in milk sold for human consumption. As with uniparous females, high-milking cows are moved to pen 10 and as milk production declines they are moved to pen 11, the low milking pen. Moving cattle between pens includes redundancy in the fresh/hospital, high-milking, and low-milking pens, essentially separating calves (pens 1 and 2), heifers (pens 3 to 5) and adult cows (pens 6 to 14). Thereafter, a cow circulates through these last six pens: 12, 13, 14, 7, 10, and 11, throughout its productive lifespan, which is about 4.8 years [11]. Moving cattle between pens was assumed to occur at a constant rate of *d*_*ij*_ from pen *i* to pen *j*. Cows are moved from one pen to another in a prescribed sequence that depends on their age (i.e., calf (*i* = 1, 2), heifer (*i* = 3, 4, 5), and adult (*i* = 6,…, 14)), their state of productivity (high milking, low milking, dry cow (*i* = 10, 11, 12), and/or state of health or fertility (breeding, pregnancy, calving, fresh/hospital (*i* = 3, …, 7)). Birth and purchase rate (*b*_*j*_), as well as culling and all-cause mortality rate (*m*_*i*_) affect the pen totals at any point in time. Following the CM model structure (Fig 1), the corresponding set of ODEs was developed as equation (1) (see S3 Appendix).

#### Estimating the between-pen movement rates

Data from four California dairies was accessed retrospectively through their dairy herd improvement software (DairyComp 305, Valley Agricultural Software, Tulare, CA) (S4 Appendix). Records from varying intervals of time between January 2011, to June 2015 were used to estimate rates of moving cows between pens. Dairy 1 contributed pen movement data from two different time periods demarcated by the dairy herd’s transition from an all Holstein to a mixed breed (Holstein and Jersey) essentially acting as two dairies and hence bringing the total to five herds.

Herd managers and veterinarians were interviewed to identify the pen types and pen population demographics including age, production and reproductive status. Although 14 pen types were identified, cattle of the same age, production, or reproductive state were housed in one or more physical pens. Hence cow movement rates were estimated for pen type and not for each pen. Pen population records from the study dairies were examined by programming a Matlab code. The code employs the optimization toolbox (with program “fmincon.m”) to estimate the time intervals and calculate each cow’s unique residence time in a pen. To account for the possibility that a cow may have been moved into a pen before the record extraction date, 5 days were added to the beginning and end of each cow’s unique record date, respectively. Subsequently, the rates of moving cows between pens were estimated as the inverse of the pen residence times for each dairy farm.

#### MAP transmission models

The progression of MAP infection is embedded into each movement model state and hence essentially in each pen’s environment. However, different MAP transmission models were specified depending on cattle age. The mathematical models included a Susceptible-Latent-Environment (SLE) model in pre-weaned calves (*i* = 1), a Susceptible-Latent-Infectious-Environment (SLIE) model for cattle from weaning to the first calving (*i* = 2, …, 6), and a Susceptible-Latent-Infectious-Super shedder -Environment (SLICE) model for older cattle (*i* = 7, …, 14). Table 2 summarizes the variables of the above-mentioned models according to the rate of fecal MAP shedding rate as described by [12].

**Table 2:** Variables of the SLE, SLIE, and SLICE models of MAP transmission.

Parameter^1^	Description
S_i_	Susceptible cattle; Cattle that have not been exposed to MAP bacteria
L_i_	Latent cattle; Cattle exposed to MAP but cannot shed the bacilli to the
	environment or transmit infection to other cattle
I_i_	Infectious cattle; Shedding less than 10,000 CFU/gr of feces^2^
C_i_	Super Shedder; Cattle shedding greater than 10,000 CFU/gr of feces^3^
N_i_	Total cattle population in pen *i*
P_i_	Number of infectious units^4^ in pen-specific environment
E	Number of infectious units^4^ in general environment

Notes

^1^ Subscript i corresponds to the pen number.

^2^ Threshold for concentration of MAP shed in feces of super-shedder cows.

^3^Colony Forming Units (CFU)/gr of feces.

^4^ i_._ Each infectious unit consists of 100 CFU.

**Fig 2:**
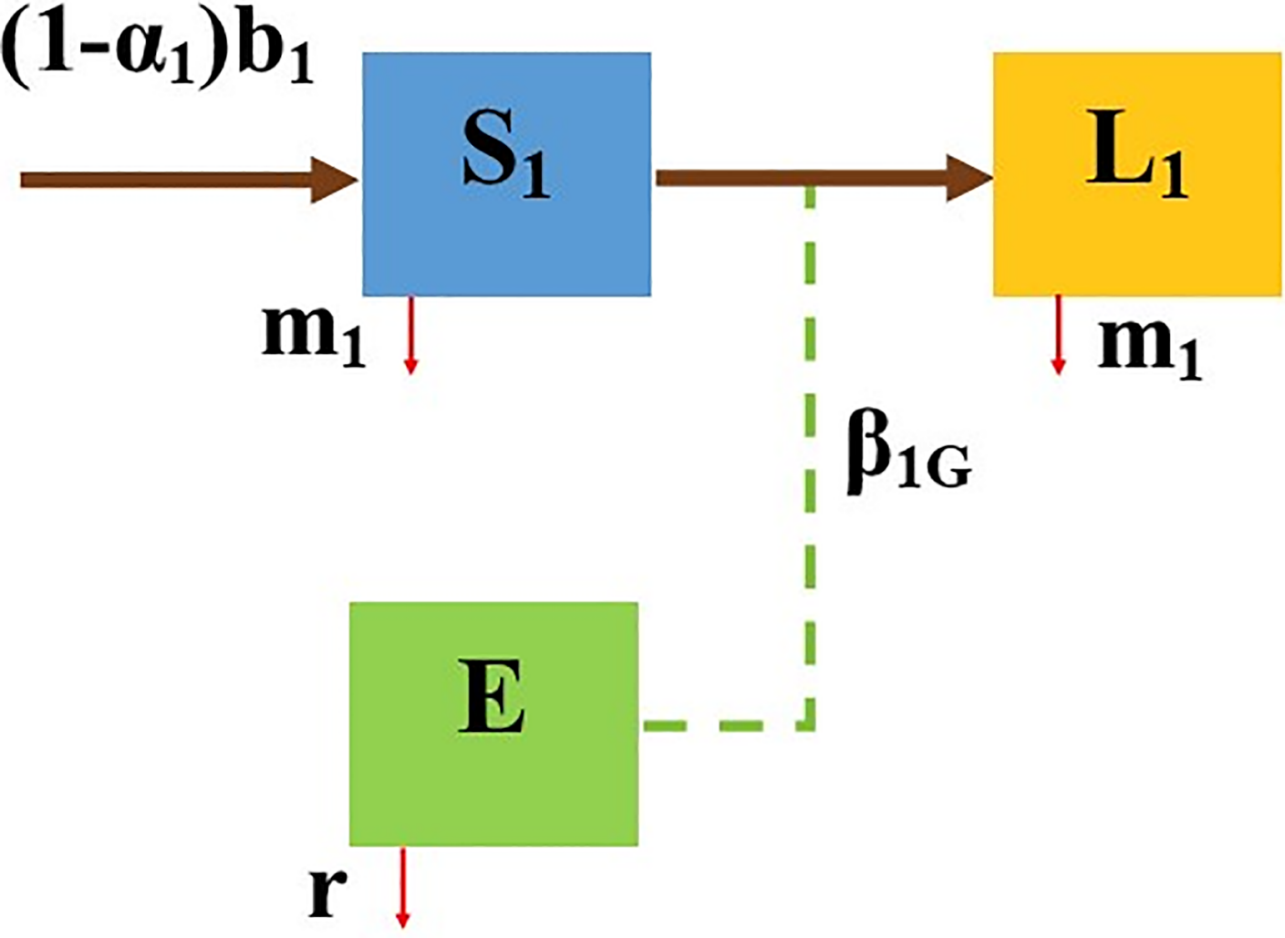
Compartmental diagram of the Susceptible-Latent-Environment (SLE) model in preweaned calves (pen type 1).

Specifically, the Susceptible (S_i_) animals in pen *i* can be exposed to the infection by direct host-to-host contact, or indirectly by contacting the contaminated pen environment (i.e., the pathogen load in barn surfaces denoted by P_i_) or the general environment (i.e., the pathogen load in the recycled lagoon water, commonly used to flush pen surfaces, denoted by E). The host-to-host contact occurs when susceptible individuals come into contact with infectious individuals including super-shedders. The exposed animals in pen *i* are infected, but yet to be infectious, hence are latent and denoted by L_i_. As infection progresses the latently infected cattle in pen *i* become infectious (I_i_) and eventually may become super-shedders (C_i_). Fig 2 depicts the MAP transmission model in preweaned calves in pen 1 of the CM model, which distinguishes between three classes: Susceptible (S_i_), Latent (L_i_), Environment (E) here onwards referred to as the SLE model and the corresponding system of ODEs (2) is given in S3 Appendix.

Intrauterine transmission from infected dams to their calves while rare, occurs even when a dam is subclinical [13]. This process has been considered in SLE model (Fig 2), where *a* is the proportion of the newborn calves that are born latently infected and 1-*a* is the remaining susceptible proportion. Although calves are presumably more susceptible to MAP infection compared to adult cattle [14], it is very difficult to identify infected calves due to the disease's prolonged latent period. Furthermore, studies suggest that MAP infected pre-weaned calves do not commonly shed the bacterium and the disease in this age group may not necessarily include an infectious state [15]. Hence, the SLE model assumes that the number of shedding calves is either negligible or the amount of shedding does not sustainably influence the transmission dynamics of JD (Fig 2). Furthermore, animals may show no clinical symptoms of disease for years after infection [16] and diagnostic tests are not sensitive enough to identify infected animals in this latent stage. Therefore, susceptible preweaned calves may progress to the latent stage but may not contaminate the pen environment (i.e., P_1_(*t*) = 0 for all *t* = 0).

**Fig 3:**
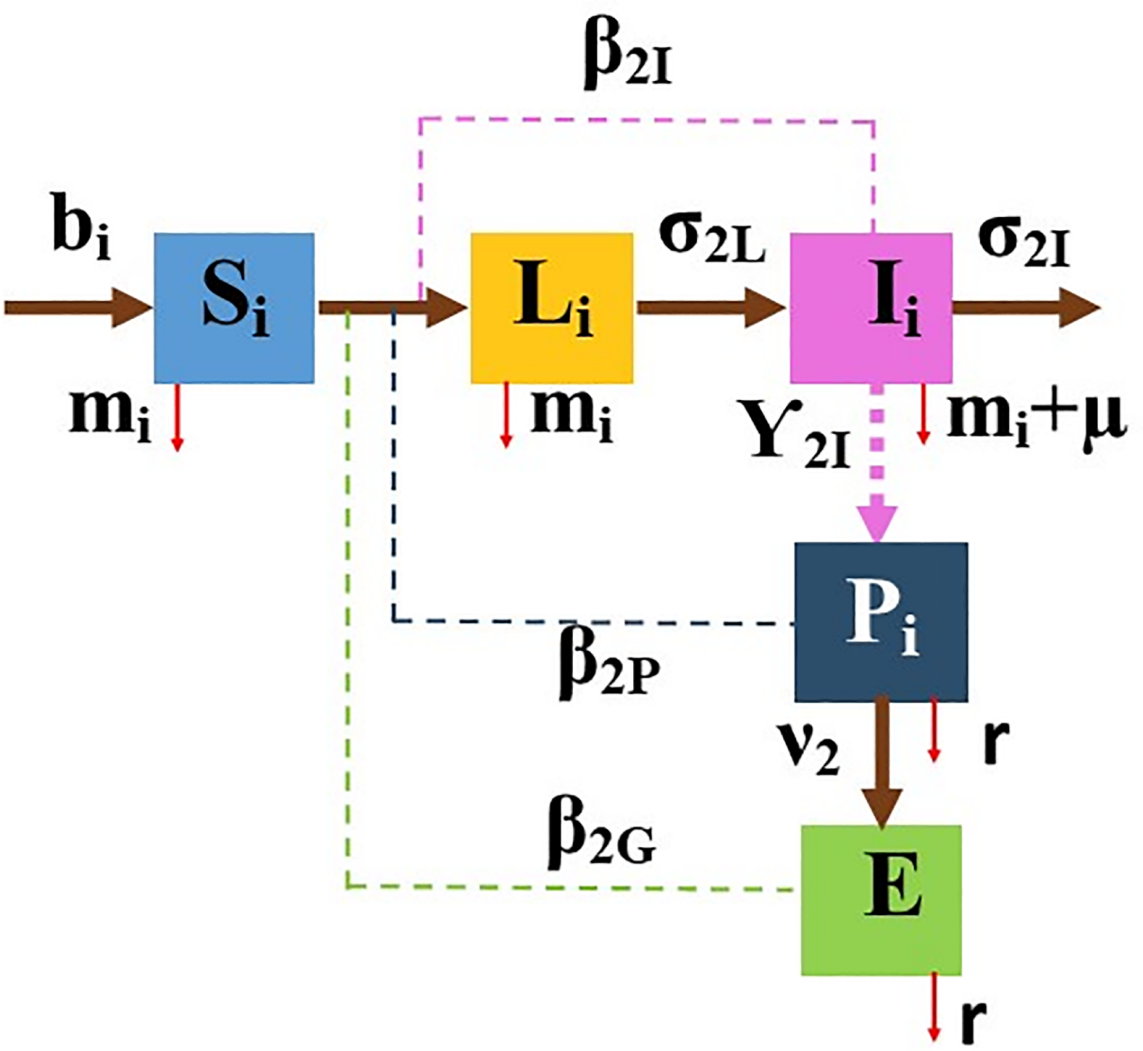
Compartmental diagram of the Susceptible-Latent-Infectious-Environment (SLIE) model in cattle from weaning to their first calving (pen types *i* = 2, …, 6). Dashed lines represent MAP shedding or transmission.

For SLIE and SLICE models, the main assumption is that a susceptible host can become infected after direct contact with contaminated environment, an infectious host or a super-shedder. Infected cattle shed the MAP bacilli into their feces and hence the pen environment, which contaminates the general environment; that infection also spreads due to the cattle moving dynamics, further exposing susceptible cattle on the dairy farm. The primary transmission route for the disease is fecal-oral [17]. Free-living MAP can survive more than a year in the environment [14]. In addition, MAP has been found in milk and colostrum [18], semen [19], blood and saliva [20]. The SLIE model is considered in pens *i* = 1, …, 6 with the assumption that calves and heifer MAP transmission do not include the super-shedding stage of MAP infection due to their younger age (Fig 3). The SLICE model is considered in pens *i* = 7, …, 14, which includes supper-shedders. See S3 Appendix for the set of ODEs (3) corresponding to the SLIE model.

#### Assembling the NC model

The progression of MAP infection at various stages of infection was incorporated within each pen of the CM model. Calf population in pen 1 is divided into susceptible and latent individuals, with disease dynamics based on the SLE model. In Fig 1, the rates *m*_*1*_ and *b*_*1*_ represent the mean culling/all-cause mortality rate, and purchase/birth rate, respectively. A proportion *a*_*1*_ of newborn calves are born latently infected and the rest (i.e., 1- *a*_*1*_) are susceptible. The coefficient *β*_*1G*_ represents the transmission rate due to exposure to MAP in the general environment including due to recycled lagoon water from the entire dairy and used to flush below the calf hutches, and 1/r is the duration of pathogen survival. Use of fresh water to flush is recommended, however recycled lagoon water collected from the parlor and flush from adult pens is sometimes used exposing calves to MAP from the remaining herd [21].

Next, we embedded the SLIE model into the CM model for pens *i* = 2, …, 6 (from weaning to calving). These pens do not include super-shedders (i.e., C_i_(*t*) = 0 for all *t* ≥ 0) due to age of the cattle. See the compartmental diagram in Fig 3 and the set of ODEs (3) in S3 Appendix, corresponding to the SLIE model.

Each of the rates *m*_*i*_, *μ*_*i*_, *b*_*i*_, for *i* = 2, …, 6, represents the mean culling and all-cause mortality rate, farm animal removal rate, and purchase/birth rate, respectively. Each of the coefficients *β*_*2I*_, *β*_*2P*_, and *β*_*2G*_ represents the transmission rate due to infectious cattle (*β*_*2I*_), pen environment (*β*_*2P*_), and the general environment due to recycled lagoon water used to flush the entire dairy (*β*_*2G*_). Each of *σ*_*2L*_, *σ*_*2I*_, *ϒ*_*2I*_, *v*_*2*_, and *r* represents infection state rates for change from the latent stage (*σ*_*2L*_), infectious (*σ*_*2I*_), mean infectious shedding (*ϒ*_*2I*_), transition rate from a pen to the general environment (*v*_*2*_), and the duration of pathogen survival (*r*).

**Fig 4:**
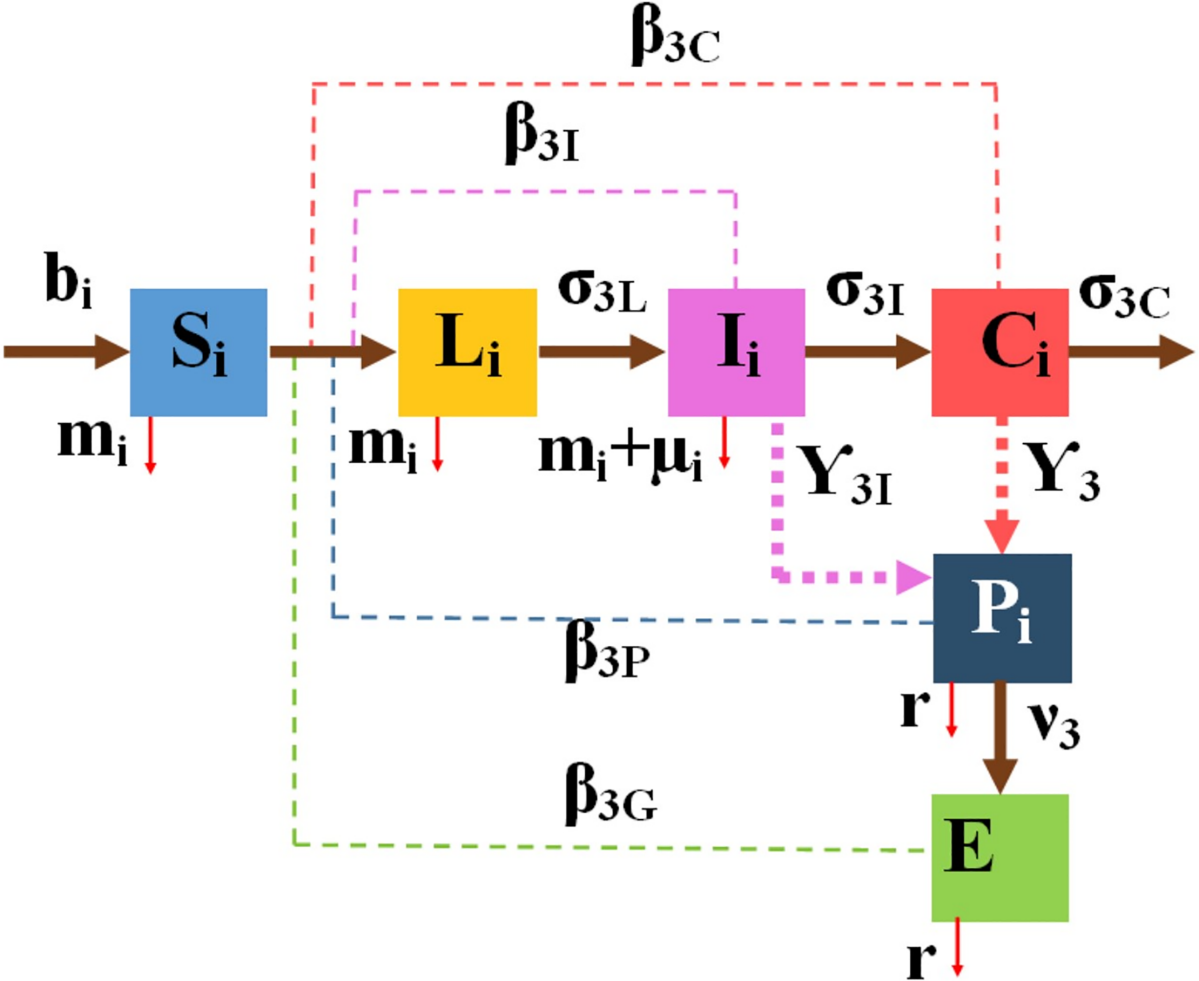
Compartmental diagram of the Susceptible-Latent-Infectious-Supper Shedder-Environment (SLICE) model in adult cattle (pen types *i* = 7, …, 14).

The SLICE model was embedded in pens *i* = 7, …, 14 (adult cattle including super-shedders C_i_(*t*) see Fig 4). Dynamics of this model is described by the system of ODEs (4) in S3 Appendix. The SLICE model represents the progression of MAP infection in the late stages of the disease which includes Tiwari et al’s [22] description of a fourth phase being the advanced clinical infection when cattle may show emaciation as body proteins are metabolized resulting in death due to cachexia. Cows are usually culled before the super-shedder state, aided by being fecal test positive with high concentrations of MAP shed in feces [22].

**Fig 5:**
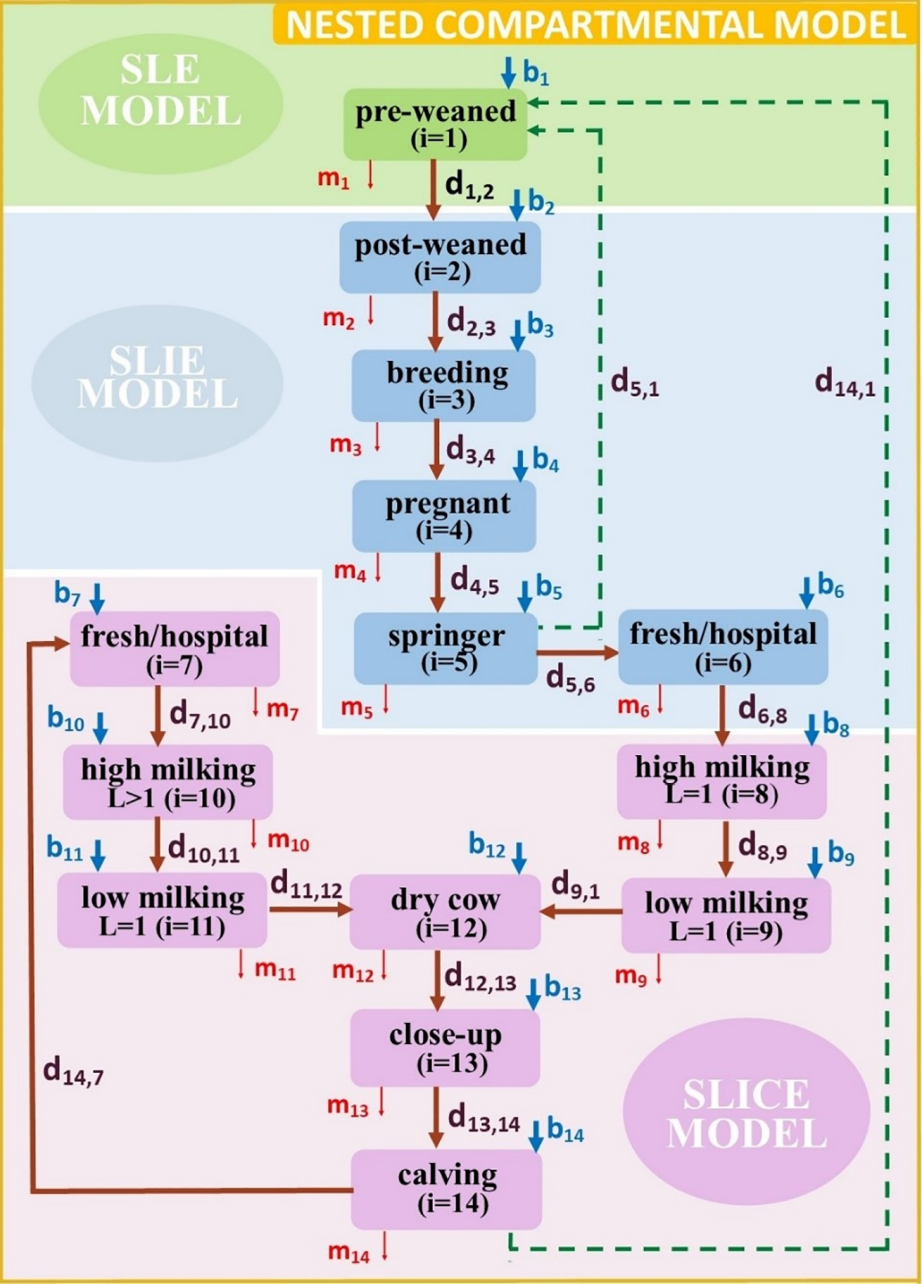
Flow chart of the NC model of MAP transmission between pens types 1, …, 14 on a cattle dairy. Models SLE, SLIE and SLICE are placed in the NC model, according to the age of the cattle.

The assembled NC model includes the CM model with the embedded MAP transmission models, SLE, SLIE and SLICE models, which is presented in Fig 5. The NC model is formulated with three systems of ODEs (5)—(7) provided in S3 Appendix.

#### Control and prevention of MAP transmission

The NC model was simulated to investigate the effectiveness of the MAP control and prevention strategies with respect to cattle movements between the pens. The main objective was to identify the optimal combination of strategies for MAP control and prevention. Five strategies for prevention and control of MAP on dairy farms were contrasted. An explanation of these measures (control 1–5) is as follows.

1 **Colostrum management:** Feeding colostrum replacer (CR) vs. maternal colostrum (MC) is expected to reduce exposure to MAP either shed in colostrum or contaminating it at harvesting, transport or feeding to newborn calves [23].
2 **Offsite heifer-rearing:** Newborn calves are removed at birth with the goal of reducing exposure of calves to MAP [24, 25]. Calves are instead raised off-site at a calf nursery with no adult cattle or recycled lagoon water and are returned to the source dairy at an older age such as post-weaning, breeding age, or as springers prior to their first calving.
3 **Intensive environmental cleaning:** MAP bioburden is reduced in the environment to decrease the natural challenge of susceptible cattle. Literature on this approach is scarce but such a measure is employed by more frequent scraping of fecal slurry in pen floors and power washing enclosure surfaces. Amongst examples of cleaning the environment of calves is the practice of cleaning hutches after weaning and before newborn calves are housed in them. Hutches are commonly cleaned by being soaking using a specially fabricated sprinkler system for hours to days before being power-washed, inverted for exposure to sunlight’s ultraviolet rays for days and on some premises hutches are then sprayed using lime, also known as liming or white washing. An example of cleaning the environment of adult cattle include the scraping and cleaning of maternity pen surface either using power washing, application of disinfectants including lime, and repacking with bedding material between calvings. In the NC model, the amount of pathogen concentration in the environment can be expressed as a function of the cleaning efficiency [26]. As mathematically shown in [5, 27] an exponential pathogen reduction such as 10-fold or more is possible for a farm that resumes/adapts a cleaning policy or uses fresh water instead of the lagoon water.
4a **Test and cull scenario (a):** This measure relies on testing dairy cows at dry off using serum enzyme-linked immunosorbent assay (ELISA) on a weekly basis and culling test- positive cows and hence affects cows in pen 12 only [28]. A sensitivity of 34.2% and specificity of 95.8% has been measured for MAP ELISA [28, 29]. We incorporated these values in the NC model simulations related to test and cull scenario.
4b **Test and cull scenario (b):** It is based on testing all the adult cows (lactating and dry) annually using serum ELISA and hence affects cows in pens 7–14 [28] using the same ELISA diagnostic accuracy as in strategy 4a.
5 **Delayed exposure:** The exposure of susceptible adult cattle to MAP infected herd mates is delayed. This is in contrast with control 2, where exposure of susceptible calf to MAP infected herd is prevented at birth. Estimates for such a measure were based on adult cow infection upon introduction to an infected herd [30].

#### The basic reproduction number *R*_*0*_

The basic reproduction number, *R*_*0*_, is defined as the mean number of secondary infections caused by a typical infected individual introduced into a totally susceptible population [6, 31]. In particular, infection will gradually disappear if *R*_*0*_ < 1; whereas, an outbreak is expected when *R*_*0*_ > 1. Due to the long incubation period, variable severity and duration of clinical disease, the term “occurrence” was used instead of “outbreak” of MAP infection. The next-generation matrix approach [6, 31, 32] was used to estimate *R*_*0*_ according to the NC model. Particularly, Infectious (I), General Environment (E), Pen Environment (P), and Super-shedder (C) compartments are considered as disease compartments, and the largest nonnegative eigenvalue of K matrix is the *R*_*0*_.

The SLE disease model of pre-weaned calves had the disease-free equilibrium (DFE) given by $\left[\begin{array}{c} S_{i}\\ L_{i}\\ E \end{array}\right]=\frac{b_{i}}{m_{i}}\left[\begin{array}{c} 1-\alpha_{1}\\ \alpha_{1}\\ 0 \end{array}\right],\;b_{1}\neq 0$ where *i = 1*. Note that the SLE model does not have any endemic equilibrium and $R_{0}^{\left[SLE\right]}{=}_{}0,$ which implies that the DFE is stable, and there will be no occurrence of disease in pen 1 at any time. This is due to the fact that there is no infectious individual considered in pen 1 and the general environment can only make the individuals latent. Another way to show the above result is through the linear stability analysis of DFE.

For the SLIE Model of MAP transmission in post-weaned, breeding, pregnant, and springer pens, the DFE and the basic reproduction number are given by

$$\begin{array}{l} {\left(S_{i},L_{i},I_{i},E\right)}_{DFE}=\left(\frac{b_{i}}{m_{i}},0,0,0,0\right)\;\mathrm{and}\\ R_{0}^{\left[SLIE\right]}=\frac{\beta_{2I}\sigma_{2L}}{\left(m_{i}+\mu_{i})(m_{i}+\sigma_{2L}\right)}+\frac{b_{i}\beta_{2P}\gamma_{2I}\sigma_{2L}}{m_{i}(r+v_{2})(m_{i}+\mu_{i})(m_{i}+\sigma_{2L})}+\frac{b_{i}\beta_{2G}\gamma_{2I}\sigma_{2L}v_{2}}{rm_{i}(r+v_{2})(m_{i}+\mu_{i})(m_{i}+\sigma_{2L})} \end{array}$$

where *i = 2*,*…*,*6*.

Similarly, for the SLICE Model of MAP transmission in adult pens, the DFE and the basic reproduction number are given by

$$\begin{array}{l} {\left(S_{i},L_{i},I_{i},C_{i},E\right)}_{DFE}=\left(\frac{b_{i}}{m_{i}},0,0,0,0\right)\;\mathrm{and}\\ R_{0}^{\left[SLICE\right]}=\frac{\beta_{3I}\sigma_{3L}}{\left(m_{i}+\sigma_{3L})(m_{i}+\mu_{i}+\sigma_{3L}\right)}+\frac{\beta_{3C}\sigma_{3I}\sigma_{3L}}{\left(m_{i}+\sigma_{3C})(m_{i}+\sigma_{3L})(m_{i}+\mu_{i}+\sigma_{3I}\right)}\\ +\frac{b_{i}\beta_{3P}(\gamma_{3I}m_{i}\sigma_{3L}+\gamma_{3C}\sigma_{3I}\sigma_{3L}+\gamma_{3I}\sigma_{3C}\sigma_{3L})}{m_{i}(m_{i}+\sigma_{3C})(m_{i}+\sigma_{3L})(r+v_{3})(m_{i}+\mu_{i}+\sigma_{3I})}+\frac{b_{i}\beta_{3G}v_{3}(\gamma_{3I}m_{i}\sigma_{3L}+\gamma_{3C}\sigma_{3I}\sigma_{3L}+\gamma_{3I}\sigma_{3C}\sigma_{3L})}{rm_{i}(m_{i}+\sigma_{3C})(m_{i}+\sigma_{3L})(r+v_{3})(m_{i}+\mu_{i}+\sigma_{3I})}. \end{array}$$

See S1 Appendix for the details of the calculations.

For the NC model, the *R*_*0*_ calculation was done numerically. Although full *R*_*0*_ expression of the NC model was not achievable, it was possible to obtain *R*_*0*_ expression according to each pen and the pens before and after it. These *R*_*0*_ expressions are listed as follows:

$$\begin{array}{l} R_{0}^{\left[1\right]}=0\\ R_{0}^{\left[2-5\right]}=\frac{\beta_{2I}\sigma_{2L}}{\left(d_{i,j+1}+m_{i}+\mu_{i})(d_{i,j+1}+m_{i}+\sigma_{2L}\right)}+\frac{\beta_{2P}\gamma_{2I}\sigma_{2L}(b_{i}+S_{i-1}d_{i-1,j})}{\left(d_{i,j+1}+m_{i})(r+v_{2})(d_{i,j+1}+m_{i}+\mu_{i})(d_{i,j+1}+m_{i}+\sigma_{2L}\right)}\\ \;+\frac{\beta_{2G}\gamma_{2I}\sigma_{2L}v_{2}(b_{i}+S_{i-1}d_{i,j})}{r(d_{i,j+1}+m_{i})(r+v_{2})(d_{i,j+1}+m_{i}+\mu_{i})(d_{i,j+1}+m_{i}+\sigma_{2L})}, \end{array}$$

where *i*, *j = 2*,*…*,*5;d*_*i*−1,*j*_ and *d*_*i*,*j*+1_ are the cattle movement rates from the pen before and to the pen after, respectively.

The remaining *R*_*0*_ expressions (pen 6–14) are listed in S3 Appendix. As mentioned above, *d*_*i*,*j*_ is the rate of moving cattle from pen *i* to pen *j*, and *i*, *j* = 1, …, 14. Let *d*_*i*,*j*_ = 0 for all pens, then all of the above-mentioned *R*_*0*_ expressions simplify such that $R_{0}^{\left[1\right]}{,}_{}\;R_{0}^{\left[2,\ldots ,6\right]},$ and $R_{0}^{\left[7,\ldots ,14\right]}$ correspond to SLE, SLIE, and SLICE models, respectively. In that case, for each *i = 2*,*…*,*14*, if $R_{0}^{\left[i\right]}>1$, then there will an occurrence of MAP. However, if $R_{0}^{\left[i\right]}<1$ for all *i* = 2, …, 14 does not guarantee that the disease will die out. Therefore between-pen cattle movements can trigger the occurrence and persistence of MAP infection on a dairy farm.

#### Global uncertainty analysis

The control measures are the strategies to control or reduce the occurrence of MAP as reflected by the basic reproduction number of the NC model. In the NC model, *R*_*0*_ has 63 parameters. To understand how *R*_*0*_ is affected by these parameters, we completed a global uncertainty analysis [33]. A total of 27 combined or individual control strategies were designed and each was simulated 50,000 times to examine their effects on prevalence and incidence of MAP infection and *R*_*0*_ value on a dairy farm of 10,000 susceptible cows with a super shedder and an infectious cow introduced to the farm. Specifically, using the range of parameter values in using the parameter values in Table 3, Tables B and C (see S3 Appendix), the numerical simulations of the NC model were carried out to estimate *R*_*0*_ values and MAP incidence and prevalence. The simulation randomly picked numbers within the given ranges for each parameter used to calculate *R*_*0*_. For each case of control strategy, 50,000 calculations of *R*_*0*_ values, minimum, maximum, mean, 95% confidence intervals (CI), and risk of MAP occurrence were calculated. The risk was calculated as the fraction of the simulation iterations, where *R*_*0*_ was greater than 1.

**Table 3:** Range of parameters values of mathematical models: Susceptible-Latent-Environment (SLE), Susceptible-Latent-Infectious-Environment (SLIE), and Susceptible-Latent-Infectious-Super Shedder Cow-Environment (SLICE) used in simulations for MAP transmission.

Notation	Description		Range of parameter values^a^			References/source	unit
			Calves: Pen 1	Heifers: Pen 2–6	Adults: Pen 7–14		
**Transmission rate**							
β_I_	infectious cattle		0	0–1.79^b^	0–3.92^c^	[2],[30],[34]	cows/yr.
β_C_	super shedder		0	0	0–3	[8],[33],[35]	1/yr.
						[36]	
β_P_	pen environment		0	0–2	0–2	Assumed	1/yr.(CFU)
β_G_	general environment		0–0.08^d^	0–0.03^e^	0–3	[8],[37]	1/yr.(CFU)
**Stage duration**							
1/σ_L_	latent^f^		N/A	0–0.33	0–0.33	[8],[38],[39]	yr.
1/σ_I_	infectious^f^		N/A	2–10	2–10	[8],[38],[39]	yr.
1/σ_C_	super shedders		N/A	2–4	2–4	[8],[38],[39]	yr.
**Environment-related**							
γ_I_	avg. shedding rate of		0	0-8e3	0-1e4	[37],[40],	CFU/yr.(cow)
	infectious					[41]	
γ_C_	avg. shedding rate of		0	0	1.26e4 –	[40]	CFU/yr.(cow)
	super shedders				1.26e6		
					CFU/g^g^		
1/r	duration of pathogen		0.8–1.5	0.8–1.5	0.8–1.5	[8]	yr.
	survival rate						
ν	transition rate from		0.03 –	0.04 –	0.50 –	Assumed	1/yr.
	pen to general		0.06	0.89	1.25		
	environment						
α	the proportion of the		0–0.15	0–0.15	0–0.17	[15],[38],	unit free
	calf that are infected					[42]	
	at birth						
μ	farm animal removal		0–0.007	0–0.007	0–0.002	[36]	1/yr.
	rate (life span of						
	animals, other						
	disease, or selling)						

^a^Parameter values were calculated per annum.

^b^The upper limit of the β_I_ range was calculated from estimates of attributable fraction in studies reported elsewhere.

^c^The upper limit of the transmission coefficient *β*_*I*_ for adult cows in pens 7 to 14 was approximated by the percent of uninfected adult cows introduced into an infected herd and, which eventually tested positive by fecal culture for MAP.

^d^The coefficient *β*_*G*_ for transmission of MAP from the general environment to calves in pen 1 (hutches) was estimated (S2 Appendix).

^e^The coefficient *β*_*G*_ for transmission of MAP from the general environment to heifers in pens 2 through 6 was estimated based on the total number of heifers that tested positive for MAP by fecal culture (S2 Appendix).

^f^10 year farm span.

^g^median = average.

Note: 1 year = 365.25 days. Further details of the approximation are given in S2 Appendix.

Table 3 includes the range parameter values used in the model simulations. In addition to S2 Appendix, details of parameter estimations are provided in the rest of this section. As mentioned before, we assumed that the main route of MAP transmission in the calf population (i.e., pen 1) is the through the general environment. Although there are some studies that consider MAP transmission from transient shedders to susceptible calves [7, 15, 33], we assumed that the number of calf-to-calf transmission in pen 1 is negligible. Furthermore, since the calf population is separated from the heifer and adult populations, the adult-to-calf transmission rates are considered zero. As suggested in [15, 38, 43], we assumed that a portion between zero to 15% (17% in adult pens) of newborn calves become infected in the fresh/hospital, maternity and calving pens (i.e., pens 7, 13 and 14). To calculate β_G_ in the calf population we used the values given in columns 2 and 3 of Table 1 in [37] (i.e., we used the prevalence of infected calves 4/329 and 1/58 in two herds during three months of study period) for β_G_ in heifer population was estimated based on the total number of heifers that tested positive for MAP by fecal culture ([37], Table 1, and Herds 1 to 8). The upper range was calculated by dividing the number of test positive heifers, 3 to 24 months of age by the total number tested [~32/1266 = 0.0256]. The estimate was assumed to be the highest annual percentage of infected heifers since it spanned a range of 12 to 21 months of follow up and for β_G_ in the adult population we used the range considered in [8].

The infectious cattle transmission rate β_I_ in the heifer population is adopted from [8]. However, β_I_ in the adult population is calculated from the values provided in columns 3 and 4 of Table 2 in [30]. The value of β_C_ in the calf and heifer population is considered zero due to the fact that the super-shedders are only considered in the adult population. The values of β_P_ may vary considerably, depending on the quality of cleaning practices (e.g., scraping and power wash). The ranges of stage durations 1/σ_L,_ 1/σ_I_ and 1/σ_C_ are mainly adopted from the previous studies [16, 38, 39]. The average shedding rate of infectious cattle in the heifer and adult population is obtained from our clinical study [12] and the work by Bolton et al. [37]. Duration of pathogen survival is considered the same for all pens. Most studies do not characterize the duration based on the host population. We therefore considered a range of 0.8 to 1.5 year as suggested by Magonbedze et al. [8]. Pathogen transmission rate from pen environments to the general environment may vary based on the age group due to the amount of manure produced by each age group. We assumed larger values for adult populations compared to heifer and calf populations. The animal removal rates vary from farm to farm, but it is known that the rates are higher in the calf and heifer populations. We adopted the same range of values used in [36].

Historical computerized dairy farm records with information on cattle IDs in each of the farms’ pens were obtained with the herd veterinarians’ and farm owners’ permission and no animals were enrolled for the purpose of the current study.

## Results

### Analysis of cow movement data

Dairy herd movement records exported from each study herd included cow identification numbers, date of record and pen location in Table A (see S3 Appendix), for a snapshot of the dairy farm data from 2011 to 2015. Some dairies had intervals of missing data for one or more pens, the latter could be due to the dairy management not utilizing such a pen or pens, or simply due to missing backups at regular intervals. The missing data was removed from the study. As summarized in Table 4, the cattle movement data of four dairy farms were used in this study. Farm 1 contributed records from 01/07/11 through 04/10/15 with data missing during the change of its breed make up between 02/01/12 through 12/31/13 interval resulting in herds 1(a) and 1(b) of Holsteins and Holstein Jersey mix breeds, respectively. Particularly, we divided the data of Farm1 into two parts and excluded the interval with missing data. Farm 2 had pen residence records from 01/07/11 through 05/26/15. At the pen level, both Farm 1 and Farm 2 had data on cows residing in pens 1 through 13, but not pen 14 due to the transitory nature of cows moved into the maternity pen at calving only. Farm 3 contributed data from 06/15/13 through 05/13/15 on cow movements between pens 2, 3, 4, 7, …, 13 with no data recorded from pens 1, 5, and 14. Farm 4 records were the most completely recorded data for the herd of 4,604 cows. The data was complete for the period from 01/18/11 through 06/02/15 and it includes cow movement data for pens 1 through 14. Farms 1 and 2 have substantially higher numbers of cows than the other farms. Using Matlab optimization toolbox the CM model (1) was fitted to the movement data of each farm. The model fitting resulted the estimated rates of moving cows between pens (*i* to *j*) on 4 dairy farms. These *d*_*i*,*j*_ rates are presented in Table B (see S3 Appendix). Tables D and E summarize the number of observations and the number of between-pen movements for all farms, respectively (S3 Appendix).

**Table 4:** The mean number of cattle of all ages on dairy farms over a 5-year period based on dairy herd movement records.

Dataset	2011	2012	2013	2014	2015	Total
Farm 1	14,433	9,540	NA^a^	12,830	10,383	47,186
Farm 2	16,016	11,288	10,535	11,155	9,819	58,813
Farm 3	NA^a^	NA^a^	5,942	5,169	4,110	15,221
Farm 4	2,496	2,359	2,311	2,407	1,902	11,475
Total	32,945	23,187	18,788	31,561	26,214	132,695

^a^Backup of the records during that period not available (NA)

#### Effectiveness of JD control measures

In summary, serum ELISA test and cull is the most effective single control measure in reducing MAP infection. By far the best outcome is obtained by combining three control measures of test and cull, cleaning, and isolating calves and heifers from the herd. The risk of MAP occurrence was calculated by dividing the number of iterations with *R*_*0*_ greater than one by total number of iterations that *R*_*0*_ has been calculated. When we compare the no control option versus all combined control strategies, the risk of MAP occurrence in dairy cattle drops from 82% to 42% and the mean *R*_*0*_ value drops from 3.92 to 0.89. Although this demonstrates a very effective approach to JD management on a dairy farm, it reveals that the MAP occurrence risk is not eliminated even though that all control measures are simultaneously applied.

Despite 42% risk of MAP occurrence, simulations of a MAP infected herd showed that employing all control measures reduced mean prevalence of MAP below 0.02% in calves and heifers, and mean prevalence in adult cows of 1.05% over ten years. Hence complete eradication of MAP was not possible, despite the fact that the prevalence and incidence of MAP were extremely low in the window of ten years.

Table 5 summarizes results of the NC model simulations assuming that each control measure separately applied to a dairy farm. S1 Fig depicts the distributions of *R*_*0*_ values. In each panel, the curve represents the fitted generalized extreme value distribution. See Table F (S3 Appendix) for the estimated sigma and mean values. In the absence of any control measures, identified as Control 0 in Table 5, the mean *R*_*0*_ value was 3.92 and with a long tail in the frequency plot such that it exceeded *R*_*0*_ = 20. The numerical simulations indicated that none of the controls were individually effective and hence they each failed to reduce the mean *R*_*0*_ values to below 1. In this regard, the top three measures were controls 4b, 3 and 4a with the mean *R*_*0*_ values of 1.31, 1.51, and 2.11, respectively.

**Table 5:** Descriptive statistics for *R*_*0*_, the basic reproduction number, for MAP transmission simulated using a NC model for single control measures applied to a dairy cattle herd.

Control measure^a^	Basic reproduction number (R_0_)				Risk of occurrence
	Mean	Min	Max	95% CI	
0	3.92	0	20.33	3.89–3.96	0.81
1	3.91	0	20.28	3.88–3.95	0.81
2	3.86	0	20.07	3.82–3.89	0.81
3	1.51	0	4.50	1.50–1.52	0.64
4a	2.11	0	7.16	2.09–2.12	0.74
4b	1.31	0	3.33	1.31–1.32	0.60
5	3.78	0	19.69	3.75–3.82	0.81

^a^Risk is the proportion of the number of times that R_0_ was greater than 1.

Control measure: 0 = No control measure; 1 = Colostrum management feeding colostrum replacer (CR) vs. maternal colostrum (MC); 2 = Offsite heifer-rearing; 3 = Reducing MAP bioburden in the environment by10-fold by scraping fecal slurry on hard surfaces or power washing; 4 = test and cull, scenario a: testing at dry off on a weekly basis and culling test-positive cows; scenario b = testing all the adult cows (lactating and dry) annually; 5 = Delaying exposure to infected cows at adult hood.

Table 6 summarizes statistics of *R*_*0*_ values for MAP transmission under all possible binary combinations of the control measures. Also, S2 and S3 Figs shows the related *R*_*0*_ distributions. Although the mean *R*_*0*_ values was reduced from 3.92 to 3.85 but the combination of controls 1 and 2 were not successful in reducing the risk of infection and hence MAP transmission. Control measure 4a, weekly test and cull of cows at dry-off (pen 12), and 4b, annual test and cull of adult cows (pens 7–14) made up the most effective binary combination control measure, while a combination of Control 3 with Control 4a or 4b was the second most effective combination control measure. Nevertheless, none of these binary combinations reduced the mean *R*_*0*_ value below one. However, as shown in Table 6 the risk significantly dropped in the cases in which test and cull (i.e., Control 4a or 4b) was combined with a control measure other than controls 1 or 2 (see S3 Appendix for more details of distribution curves and the related probability density functions). In the best case scenario, the risk of MAP occurrence decreased from 81% (control 0) to 47% (controls 4a and 4b). Also, in all cases the mean R_0_ value was greater than 1, which indicates that JD will gradually spread in the herd.

**Table 6:** Descriptive statistics for *R*_*0*_, the basic reproduction number, for MAP transmission simulated using the NC model for binary combinations of control measures in a dairy cattle herd.

Control measure^a^	Basic reproduction number (R_0_)				Risk of occurrence
	Mean	Min	Max	95% CI	
1 & 2	3.85	0	20.05	3.82–3.89	0.81
1 & 5	3.78	0	19.64	3.74–3.81	0.81
2 & 5	3.78	0	19.69	3.75–3.82	0.81
1 & 4a	2.10	0	7.14	2.09–2.11	0.73
2 & 4a	2.06	0	7.00	2.05–2.08	0.73
3 & 5	2.02	0	6.83	2.00–2.03	0.72
5 & 4a	2.02	0	6.83	2.00–2.03	0.72
1 & 3	1.50	0	4.49	1.49–1.50	0.63
2 & 3	1.49	0	4.49	1.48–1.50	0.63
1 & 4b	1.30	0	3.32	1.29–1.31	0.59
2 & 4b	1.29	0	3.31	1.28–1.30	0.59
5 & 4b	1.27	0	3.28	1.27–1.28	0.59
3 & 4b	1.21	0	3.01	1.21–1.22	0.56
3 & 4a	1.09	0	2.52	1.09–1.10	0.51
4a & 4b	1.01	0	2.22	1.01–1.02	0.47

^a^See the description of control measures in the footnote of Table 5.

**Fig 6:**
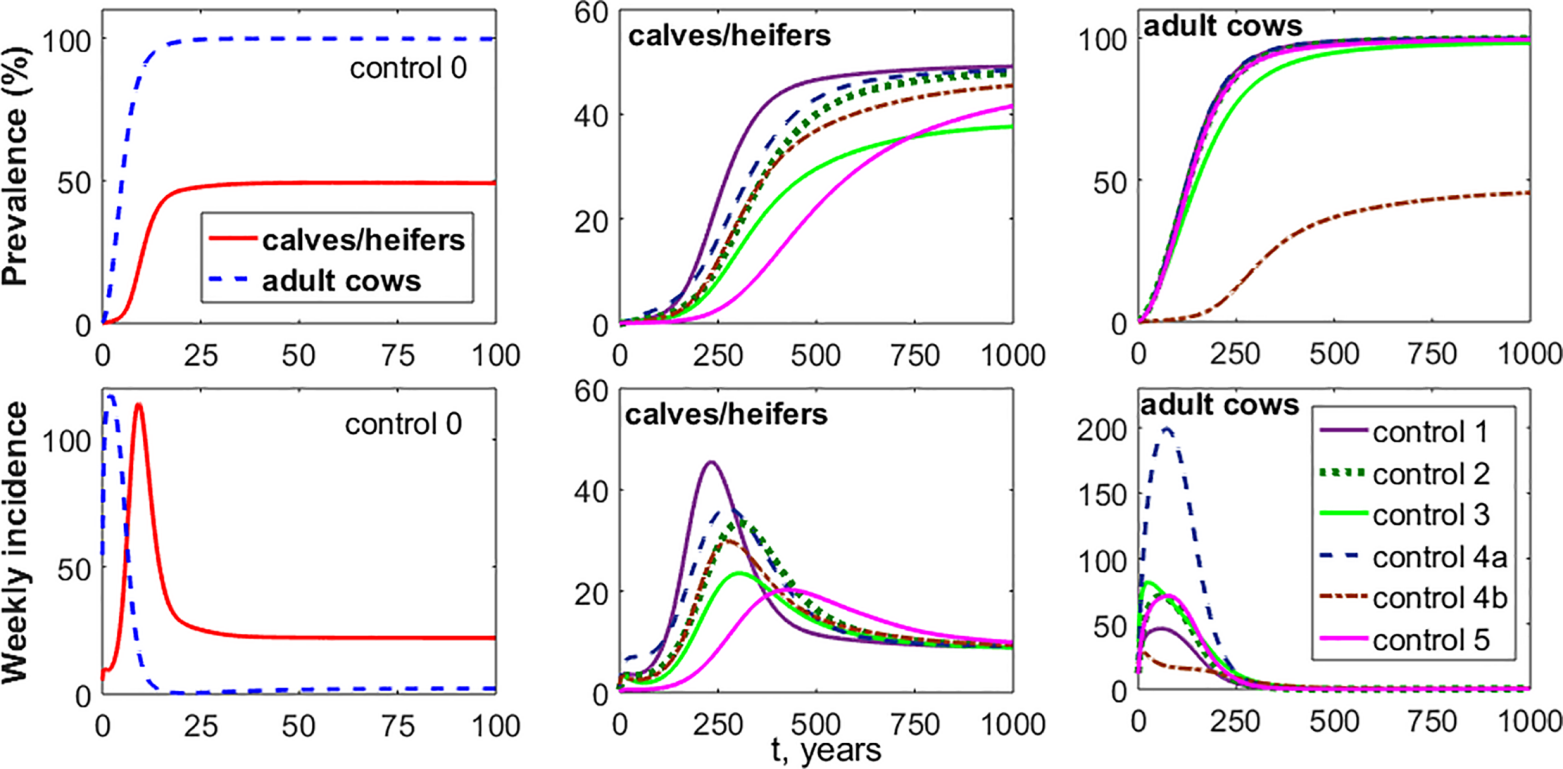
Asymptotic behavior of MAP prevalence and incidence simulated with the NC model. The curves are the mean values of 50,000 simulations. The first column corresponds to a dairy cattle herd without any implemented control measure (control 0). The second and third columns correspond to a dairy cattle herd under single control measures 1–5. Despite significant reductions/slowdowns in the incidence and prevalence, none of the single control measures were capable of eradicating the infection.

Although risk of MAP infection decreased with triple and all control measures, the risk was not eliminated and remained non-zero. In particular, as shown in Table 7, the risk of MAP infection decreased from 81% (control 0) to 42% (controls 3, 4a and 4b) and the mean *R*_*0*_ value decreased from 3.92 to 0.89. From the most effective to the least, the combined measures with mean *R*_*0*_ values less than 1 are controls All, (3 & 4a & 4b), (5 & 4a & 4b), and (2 & 4a & 4b), with mean *R*_*0*_ values of 0.898, 0.94, 0.95, and 0.97, respectively (Table 7; see also S2 Fig). Hence, on average, a farm that employs all control measures, or a combination of the three control measures should remain or gradually become disease free. However, there is still more than 40% risk of MAP infection remaining in the herd. In a similar manner, prevalence and incidence of MAP infection were estimated based on simulations for applying single control measures. Fig 6 represents the asymptotic behavior of MAP infection prevalence and incidence in a dairy farm. Namely, the curves were obtained by taking the mean values of 50,000 NC model simulations for a long period of time (*t* = 1,000 years).

**Table 7:** Min, max, mean, risk, 95% CI for best triple and all combinations of control measures.

Control measure^a^	Basic reproduction number (R_0_)				Risk of occurrence
	Mean	Min	Max	95% CI	
2 & 4a & 4b	0.97	0	2.17	0.96–0.98	0.45
5 & 4a & 4b	0.95	0	2.15	0.94–0.96	0.45
3 & 4a & 4b	0.94	0	2.04	0.93–0.95	0.42
All	0.898	0	2.03	0.89–0.91	0.42

^a^See the description of control measures in the footnote of Table 5.

For each simulation, a super shedder and an infected cow are initially introduced to the herd. It can be seen that control 4b (i.e., annual test and cull of adult cows) was the most successful method in the population of adult cows. Control 3 (i.e., intensive environmental cleaning) could effectively slow down the increase of incidence and prevalence in calf and heifer populations. It should also be noted that control 5, which was designed for delaying the exposure of calves and heifers to infected cows, was the most effective method in the calf and heifer population to keep both prevalence and incidence low. This is despite the fact that control 5 had a poor efficacy of 81% risk of MAP occurrence (Table 5).

Details of the asymptotic values associated with the single measures can be found in Table F (S3 Appendix). Further simulations indicated that a combination of test and cull (i.e. control 4a or 4b) with control 1 (i.e. Colostrum management) did not reduce the incidence and prevalence in the calf and heifer populations due to the fact that test and cull is rarely applied to calf and heifer pens.

We also calculated the range of the mean prevalence and incidence estimates in a practical time period (i.e., in the interval of 10 years) using 50,000 NC model simulations. These values are presented in Table 8, where control 5 is still the best control measure in the population of calves and heifers. In the population of adult cows there was no single control measure, which was superior to all other measures.

**Table 8:** Mean range weekly incidence and prevalence (%) of MAP transmission when single control measures are applied for a period of 10 years.

Control measures^a^	Prevalence (%) 0–10 yrs.		Number of weekly incidence 0–10 yrs.	
	Calves and Heifers: Pen 1–6	Adults: Pen 7–14	Calves and Heifers: Pen 1–6	Adults: Pen 7–14
	Mean range^b^	Mean range	Mean range	Mean range
0	0–20.63	0.26–87.74	0.08–10.39	1.54–12.71
1	0–0.33	0.19–1.09	0–0.13	0.06–0.34
2	0–0.08	0.25–1.07	0–0.03	0.09–0.48
3	0–0.32	0.28–1.13	0–0.005	0.001–0.01
4a	0–0.36	0.32–1.27	0–0.006	0.001–0.01
4b	0–0.11	0.65–1.65	0–0.04	0.06–0.13
5	0–0.008	0.29–1.22	0–0.003	0.10–0.40

^a^See the description of control measures in the footnote of Table 5.

^b^Mean range indicates the range of mean values of 50,000 NC model simulations for each of controls 0–5 in the interval of 10 years.

Hence, we investigated the prevalence and the incidence for the cases that more than one measure was employed. Namely, the results of combined control measures are presented in Table 9 showing that the mean prevalence and incidence estimates were substantially less in the calf and heifer populations. Moreover, the binary controls 3 and 4 are ineffective in calf and heifer populations, but they are effective in adult populations. NC model simulations for calves and heifer populations with the combination of double control measures 1 & 5, 2 & 5, 3 & 5, 5 & 4a, 5 & 4b (Table 9), result in prevalence estimates below 0.01% (one out of 10,000 cows), which is an important result that a disease-free herd can remain disease free, under two assumptions. First, extremely low number of infectious cow or supper shedder (*n* < 5) are accidently introduced to the herd.

**Table 9:** Mean weekly incidence and prevalence (%) of MAP transmission when binary control measures are applied for a period of 10 years.

Control measures^a^	Prevalence (%) 0–10 yrs.		Number of weekly incidence 0–10 yrs.	
	Calves/heifers	Adults:	Calves/heifers:	Adults:
	Pen 1–6	Pen 7–14	Pen 1–6	Pen 7–14
	Mean range^b^	Mean range	Mean range	Mean range
1 & 2	0–0.06	0.60–1.52	0–0.02	0–0.02
1 & 3	0–0.27	0.25–1.11	0–0.10	0.08–0.39
1 & 4a	0–0.32	0.28–1.22	0–0.13	0.06–0.18
1 & 4b	0–0.33	0.18–1.04	0–0.11	0.06–0.30
1 & 5	0–0.01	0.26–1.15	0–0.01	0.08–0.36
2 &3	0–0.06	0.60–1.49	0–0.02	0.06–0.13
2 & 4a	0–0.05	0.25–1.12	0–0.02	0.08–0.41
2 & 4b	0–0.07	0.20–1.04	0–0.02	0.11–0.56
2 & 5	0–0.02	0.24–1.10	0–0.01	0.06–0.49
3 & 4a	0–0.36	0.26–1.17	0–0.13	0.09–0.43
3 & 4b	0–0.35	0.28–1.13	0–0.13	0.06–0.24
3 & 5	0–0.02	0.33–1.18	0–0.01	0.08–0.43
4a & 4b	0–0.07	0.23–1.11	0–0.03	0.12–0.38
5 & 4a	0–0.01	0.26–1.15	0–0.004	0.08–0.36
5 & 4b	0–0.01	0.19–1.03	0–0.004	0.06–0.30

^a^See the description of control measures in Table 5.

^b^ Mean range indicates the range of mean values of 50,000 NC model simulations for each case of binary controls 1&2–5&4b in the interval of 10 years.

Second, a combination of the above-mentioned control measures is strictly implemented. A common control measure among these effective combinations is control measure 5 under which calves are born and raised in uninfected herds delaying to exposure to infected cattle. Despite being a different scenario, such Estimates may serve as a conservative (worst case) scenario resulting in a prevalence of 0.008% (less than 1 in 10,000) estimated in the heifer population, making it the most effective measure in this age group. The next most effective control measure in calves and heifers were combinations with controls 2 (i.e., 1 & 2, 2 & 3, 2 & 4a, and 2 & 4b) resulting in a prevalence of 0.07% (7 out of 10,000). All of these combinations included control 2 (off-site heifer rearing) where exposure of calves to MAP infection is avoided starting at birth by relocated them off-site before being returned to their source herd as springers. Although the offsite nursery prevents contact between the calves and adult cattle, the environment in the off-site nursery pens may be contaminated by lagoon water in case of recycling flush water; and therefore, a mean of 0.07% prevalence is expected. Nevertheless, the simulations suggested that control 2 was the second best measure to reduce the JD prevalence in the calf and heifer populations.

The mean incidence and prevalence values were extremely low due to the fact that the model simulations assumed that only one super-shedder adult cow and another infected adult cow were introduced into the herd in pen 10 and pen 8, respectively and followed for 10 years. Similar results were obtained when small numbers (i.e. *n* ≤ 5) of infected cows and super-shedders were introduced into a herd of 10,000 cows. This indicates that the illustrated results are consistent for small numbers of infectious cows and supper shedders initially introduced to the herd.

In the population of adult cows, controls 2 & 4a, 2 & 4b, 3 & 4b, 5 & 4b, 4a & 4b (Table 9), and all controls combined (Table 10) result in a MAP prevalence of 0.52%. Measures 4a (weekly test and cull of dry cows) or 4b (annual test and cull of adult cattle) are common to all the adult cattle effective control measures. Hence an effective way to reduce MAP prevalence in the adult cow population is test and cull of test-positive cattle. However, control 4a was more effective than 4b (Table 8) resulting in a MAP prevalence of 0.61% and 0.98%, respectively.

**Table 10:** Mean weekly prevalence and incidence (%) of MAP transmission when triple and all control measures are applied for a period of 10 years.

Control measure^a^	Prevalence (%) 0–10 yrs.		Weekly incidence 0–10 yrs.	
	Calves and heifers: Pen 1–6	Adults: Pen 7–14	Calves and heifers: Pen 1–6	Adults: Pen 7–14
	Mean range	Mean range	Mean range	Mean range
2 & 4a & 4b	0–0.05	0.31–1.14	0–0.02	0.08–0.25
3 & 4a & 4b	0–0.07	0.23–1.01	0–0.024	0.12–0.65
5 & 4a & 4b	0–0.01	0.41–1.35	0–0.004	0.07–0.23
All	0–0.009	0.23–1.04	0–0.004	0.11–0.29

^a^See the description of control measures in Table 5.

Table 10 shows the number of weekly incidence and the mean MAP prevalence for the most effective triple combination control measures and all of the control measures by the end of year 10 for calves and heifers were 5 & 4a & 4b (up to 0.01%); and for adult cows is seen with 3 & 4a & 4b (up to 1.01%). Simulating all the control measures results in the mean MAP prevalence by year 10 in calves and heifers of 0.009% (less than one in 10,000) and in adult cows 1.04%.

## Conclusions

The simulation results indicate that no single control measure was sufficient to prevent increase in incidence of JD; however, Control 4b (i.e., test and cull of adult cattle in a dairy herd annually) resulted in the best single control measure. The most effective combination of binary control measures was produced by controls 4a annual test and cull of adult cows (pens 7–14) and 4b (i.e., weekly test and cull of cows at dry-off cows, pen 12).

The overall risk of MAP occurrence was substantially reduced when test and cull was combined with intensive enclosure cleaning to reduce MAP concentration in the environment. Particularly, the best triple control measures resulted when combining Controls 3, 4a and 4b, which combined increased scraping of fecal slurry on solid surfaces in the dairy and /or power washing by 10-fold to reduce the environmental pathogen load, while also testing and culling dry-off cows on weekly basis and adult cattle annually. A farm that employs all control measures or a combination of these three control measures has the minimum risk of JD occurrence. It also has extremely prevalence and incidence provided that the number of infectious cow and supper shedder added to the herd is very small (i.e., less than 5 in a population of 10,000 cows).

Finally, it should be noted that these results can be expected if the dairy manager adheres to a cattle movement pattern between pens which maintains a degree of isolation between calves and cows and within the cow population as illustrated in the Cattle Movement diagram. Purposefully moving cattle between pens in a prescribed sequence changes the contact patterns between susceptible and infected cows beyond the assumption of random mixing inherent in infectious disease models. Cattle movement management is integral to the effectiveness of MAP control measures and changes to this system can modify the anticipated success of the control measures.

### Limitations of the study

Modeling JD with effects of vaccination has been addressed in previous works (see for example [15, 33]). In the present study, we did not investigate the effects of vaccination in our modeling and numerical simulations. Vaccination has its own shortcomings and is not practiced on several dairy farms. Previous research has shown that exposure to MAP vaccines or M. avium antigens can result in false positive tuberculosis tests, which is a concern for herds in TB free states and specifically those that commonly transport cattle across state lines [43, 44, 45, 46]. Furthermore, no vaccine has been developed to fully protect calves [33]. There is currently no available approved treatment in food animals once an animal has contracted the MAP infection. For such reasons vaccinating against MAP is not widely practiced and hence was not considered in the current model.

In the present work we assumed that the amount of shedding in the calf population does not sustainably influence the transmission dynamics of JD, i.e., γ_C_ = 0 for pen 1 (Table 3). Nevertheless, this could be oversimplifying assumption in cases that the shedding rate is greater than a critical value.

Although the simulation result indicate that test and cull can be an effective control measure, there are two major concerns regarding test and cull. First, test and cull result is an immediate economic loss, which may not be recovered for a long period. Second, diagnostic tests to identify infected cows (e.g., ELISA-based JD control) often have low sensitivities and are often costly to apply routinely. Therefore, the efficacy of test and cull substantially varies based on the frequency and sensitivity of the test. There are simulating models [14, 33, 47] and field [16, 48] studies that aim to determine the optimal culling rate in different herds based on the long-term profitability of the control measure. However, more data and model simulations are needed to develop reliable, effective and profitable JD control programs.

It should be noted that the data related to this study is from California dairies. Hence, the outcomes of current study may not necessarily apply to non-intensive dairy systems elsewhere in the US and the world. However, for dairies that manage cows in housing units and groups similar to the study dairies our findings may apply in terms of effectiveness of control measures and what may be expected in reduction of MAP transmission. Another limitation of the current study as with other mathematical modeling studies and specifically those modeling MAP transmission is the lack of precise transmission rates and other inputs needed by the model. Such model inputs require specifically designed studies that can limit variability and target the specific rate of interest. However, MAP’s chronicity increases the duration of such studies which may translate to increase in cost in addition to prolonged duration of studies and potential for loss of follow up of study animals given other competing risks. To address these limitations, the current study identified several key assumptions that can be justified to utilize ranges of transmission rates from previous works (Table 4 and S2 Appendix).

### Additional remarks

Investigating the optimal use of the cattle movement model with additional controls can benefit from these findings as the data shows that test and cull strategies seem to give the best outcome for *R*_*0*_. When test and cull is applied in pens 7 through 14 we see the most desirable outcome. While the primary goal of this work was to determine the efficacy of control measures using a NC model applied to JD on dairy farms, such models could also be employed to explore impacts on other animals and potentially applied to other diseases.

## Supporting information

S1 Fig Frequency distribution histograms of the basic reproduction number *R*_*0*_, and fitted generalized extreme value distribution curves. The top six panels correspond to single control measures 1–5 and the bottom panel relates to control 0 (i.e., a farm without any implemented control measure). The *R*_***0***_ values were calculated with 50,000 runs of the NC model. Controls 3, 4a and 4b resulted in substantially less *R*_***0***_ values of 1.51, 2.11, 1.31 and the calculated risks of 0.64, 0.74, and 0.60%, respectively. Note that control 4b (test and cull all the adult cows (lactating and dry) annually) is the most effective single control measure.
(TIF)

S2 Fig *R*_*0*_ frequency distributions related to various binary control measures and the case of control 0 (i.e., a farm without any implemented control measure). A combination of controls 4a & 4b (4a is testing at dry off on a weekly basis and culling test-positive cows; 4b is test and cull all the adult cows (lactating and dry) annually), was the most effective binary control measure with the descriptive statistics of *R*_***0***_ values of 1.01 and the calculated risks of 0.47%.
(TIF)

S3 Fig *R*_*0*_ frequency distributions of combined control measures. In all cases, the estimated *R*_***0***_ values exceed one, which indicates that the risk of infection remains greater than zero even though that all control measures have been implemented. See Table 7 for descriptive statistics of *R*_***0***_ values and the calculated risks.
(TIF)

S1 Appendix *R*_*0*_ Calculations. (DOCX)

S2 Appendix Details of the upper limit calculation of the transmission rate. (DOCX)

S3 Appendix Supplementary Information including Summary of data collection and model formulations (Table A), *R*_*0*_ expressions for the NC model (pen 6–14), Estimated parameter values of the CM model (Tables B-C), Number observations and between-pen movements (Tables D-E), and Estimated sigma and mean values with 95% CI (Table F).
(DOCX)

S4 Appendix Supplementary data files—Farm 1a, 1b, 2, 3, 3 data. (XLSX)

## References

[1] Collins M, Manning E. What is Johne's disease and what causes it? 2010. Retrieved from http://www.Johnes.org/general/faqs.html

[2] Lovell R, Levi M, Francis J. Studies on the survival of Johne's bacilli. Journal of Comparative Pathology and Therapeutics. 1944;54: 120–129.

[3] Larsen AB, Merkal RS, Vardaman TH. Survival time of mycobacterium paratuberculosis. American Journal Veterinary Research. 1956;17: 549–551. 13340126

[4] Mitchell RM, Whitlock RH, Grohn YT, Schukken YH. Back to the real world: Connecting models with data. Preventive Veterinary Medicine. 2015;118: 215–225. 10.1016/j.prevetmed.2014.12.009 25583453

[5] Bani-Yaghoub M, Gautam R, Döpfer D, Kaspar CW, Ivanek R. Effectiveness of environmental decontamination in control of infectious diseases. Epidemiology & Infection. 2012;140(3): 542–553. 21676360 10.1017/S0950268811000604

[6] Diekmann O, Heesterbeek JAP. Mathematical epidemiology of infectious diseases. West Sussex, England: John Wiley & Sons; 2000.

[7] Lu Z, Shukken YH, Smith RL, Mitchell RM, Grohn YT. Impact of imperfect Mycobacterium avium subsp. paratuberculosis vaccines in dairy herds: a mathematical modeling approach. Preventative Veterinary Medicine. 2013b;108: 148–158. 10.1016/j.prevetmed.2012.08.001 22921715

[8] Magombedze G, Ngonghala CN, Lanzas C. Evaluation of the”Iceberg Phenomenon” in Johne’s disease through mathematical modelling. Plos One. 2013;8(10): 1–11. 10.1371/journal.pone.0076636 PMC3805542 24167547

[9] Heffernan C, Thomson K, Nielsen L. Livestock vaccine adoption among poor farmers in Bolivia: remembering innovation diffusion theory. Vaccine. 2008;26(19): pages. 2433–2442. 10.1016/j.vaccine.2008.02.045 18423805

[10] USDA. Dairy 2007, Part II: changes in the U.S. dairy cattle industry, 1991–2007. Washington DC: Animal and Plant Health Inspection Service. 2008. Available from: https://www.aphis.usda.gov/animal_health/nahms/dairy/downloads/dairy07/Dairy07_dr_PartII_rev.pdf

[11] Tijdschr DT. Productive life-span of dairy cows and its economic significance. II. The replacement of dairy cows: an economic model (J. A. Renkema & J. Stelwagen, Trans.). PubMed. 1977;102(12): 739–47. http://www.ncbi.nlm.nih.gov/pubmed/867405. 867405

[12] Aly SS, Anderson RJ, Whitlock RH, Fyock TL, McAdams SC, Byrem TM. Cost- effectiveness of diagnostic strategies to identify Mycobacterium avium subspecies paratuberculosis super-shedder cows in a large dairy herd using antibody enzyme- linked immunosorbent assays, quantitative real-time polymerase chain reaction, and bacterial culture. Journal of Veterinary Diagnostic Investigation. 2012;24(5): 821–832. 10.1177/1040638712452107 22807510

[13] Whittington RJ, Windsor PA. In utero infection of cattle with mycobacterium avium subsp. paratuberculosis: A critical review and meta-analysis. The Veterinary Journal. 2009;179(1): 60–69. 10.1016/j.tvjl.2007.08.023 17928247

[14] Martcheva M, Lenhart S, Eda S, Klinkenberg D, Momotani E, Stabel J. An immuno- epidemiological model for Johne’s disease in cattle. Veterinary Research. 2015;46: 69 10.1186/s13567-015-0190-3 26091672 PMC4474574

[15] Lu Z, Shukken YH, Smith RL, Grohn YT. Using vaccination to prevent the invasion of Mycobacterium avium subsp. paratuberculosis in dairy herds: a stochastic simulation study. Preventative Veterinary Medicine. 2013a;110: 335–345. 10.1016/j.prevetmed.2013.01.006 23419983

[16] Nielsen SS, Ersboll AK. Age at occurrence of mycobacterium avium subspecies paratuberculosis in naturally infected dairy cows. Journal of Dairy Science. 2006;89(12): 4557–4566. 10.3168/jds.S0022-0302(06)72505-X 17106087

[17] Sweeney RW. Transmission of paratuberculosis. The Veterinary Clinics of North America. Food Animal Practice. 1996;12(2): 305–12. 8828107 10.1016/s0749-0720(15)30408-4

[18] Streeter RN, Hoffsis GF, Bech-Nielsen S, Shulaw WP, Rings DM. Isolation of mycobacterium paratuberculosis from colostrum and milk of sub clinically infected cows. American journal of veterinary research. 1995;56(10): 1322–1324. 8928949

[19] Ayele WY, Bartos M, Svastova P, Pavlik I. Distribution of mycobacterium avium subsp. paratuberculosis in organs of naturally infected bull-calves and breeding bulls. Veterinary Microbiology. 2004;103: 209–217. 10.1016/j.vetmic.2004.07.011 15504592

[20] Sorge US, Kurnick S, Sreevatsan S. Detection of mycobacterium avium subspecies paratuberculosis in the saliva of dairy cows: a pilot study. Veterinary Microbiology. 2013;164(3–4): 383–386. 10.1016/j.vetmic.2013.02.021 23517764

[21] Aly SS, Thurmond MC. Evaluation of Mycobacterium avium subsp paratuberculosis infection of dairy cows attributable to infection status of the dam. Journal of the American Veterinary Medical Association. 2005;227 (3), pp. 450–454. . 16121613 10.2460/javma.2005.227.450

[22] Tiwari A, VanLeeuwen JA, McKenna SLB, Keefe GP, Barkema HW. Johne’s disease in Canada. Part I: clinical symptoms, pathophysiology, diagnosis, and prevalence in dairy herds. The Canadian Veterinary Journal. 2006;47: 874–882. 17017652 PMC1555680

[23] Pithua P, Godden SM, Wells SJ, Oakes MJ. Efficacy of feeding plasma-derived commercial colostrum replacer for the prevention of transmission of Mycobacterium avium subsp paratuberculosis in Holstein calves. J Am Vet Med Assoc. 2009;234: 11671176. 10.2460/javma.234.9.1167 19405889

[24] Aly SS, Gardner I A, Adaska JM, Anderson RJ. Off-site rearing of heifers reduces the risk of Mycobacterium avium ssp. Paratuberculosis ELISA seroconversion and fecal shedding in a California dairy herd. Journal of Dairy Science. 2015;98(3): 1805–1814. 10.3168/jds.2014-8759 25597977

[25] Aly SS, Gardner IA, Adaska JM, Anderson R J. Off-site rearing of heifers reduces the risk of Mycobacterium avium ssp. paratuberculosis ELISA seroconversion and fecal shedding in a California dairy herd. Journal of Dairy Science. 2015;98(3): 1805–1814. 10.3168/jds.2014-8759 25597977

[26] Gautam R, Lahodny G, Bani-Yaghoub M, Morley PS, Ivanek R. Understanding the role of cleaning in the control of Salmonella Typhimurium in grower-finisher pigs: a modelling approach, Epidemiol Infect. 2014;142 (5): 1034–1049 10.1017/S0950268813001805 23920341 PMC9151178

[27] Bani-Yaghoub M, Gautam R, Ivanek R, van den Driessche P, Shuai Z. Reproduction numbers for infections with free-living pathogens growing in the environment, Journal of Biological Dynamics. 2012;6(2): 923–940. 22881277 10.1080/17513758.2012.693206

[28] Aly SS, Anderson RJ, Whitlock RH, Adaska JM. Sensitivity and Specificity of Two Enzyme-linked Immunosorbent Assays and a Quantitative Real-time Polymerase Chain Reaction for Bovine Paratuberculosis Testing of a Large Dairy Herd. Intern J Appl Res Vet Med. 2014;12(1): 1–7.4.

[29] Angelidou E, Kostoulas P, Leontides L. Bayesian estimation of sensitivity and specificity of a commercial serum/milk ELISA against the Mycobacterium avium subsp. Paratuberculosis (MAP) antibody response for each lactation stage in Greek dairy sheep. Prev Vet Med. 2016;124: 102–5. 10.1016/j.prevetmed.2015.12.011 26754926

[30] Espejo LA, Kubat N, Godden SM, Wells S J. Effect of delayed exposure of cattle to Mycobacterium avium subsp paratuberculosis on the development of subclinical and clinical Johne’s disease. American Journal of Veterinary Research. 2013;74(10): 1304–1310. 10.2460/ajvr.74.10.1304 24066914

[31] Van Den Driessche P, Watmough J. Reproduction numbers and subthreshold endemic equilibria for compartmental models of disease transmission. Math. Biosci., 2002;180: 29–48. 12387915 10.1016/s0025-5564(02)00108-6

[32] Chowell G, Brauer F. The basic reproduction number of infectious diseases: computation and estimation using compartmental epidemic models In: Chowell G, Hyman JM, Bettencourt LM, Castillo-Chavez C, editors. Mathematical and statistical estimation approaches in epidemiology. New York, NY: Springer; 2009 Pages 1–30.

[33] Lu Z, Mitchell RM, Smith RL, Van Kessel JS, Chapagain PP, Schukken YH, et al The importance of culling in Johne’s disease control. J. Theor. Biol. 2008;254: 135–146. 10.1016/j.jtbi.2008.05.008 18573505

[34] Pithua P, Wells SJ, Godden SM. Evaluation of the association between fecal excretion of Mycobacterium avium subsp paratuberculosis and detection in colostrum and on teat skin surfaces of dairy cows. Journal of the American Veterinary Medical Association. 2011;238(1): pp. 94–100. 10.2460/javma.238.1.94 21194328

[35] Cho J, Tauer L, Schukken YH, Smith RL, Lu Z, Grohn YT. Cost-effective control strategies for Johne’s disease in dairy herds. Canadian Journal of Agricultural Economics. 2013;61(4): 583–608.

[36] Massaro T, Lenhart S, Spence M, Drakes C, Yang G, Agusto F, et al Modeling for cost analysis of Johne’s disease control based on EVELISA testing. J Biol Syst. 2013;21: 1340010 10.1142/S021833901340010X

[37] Bolton MW, Pillars RB, Kaneene JB, Mauer WA, Grooms DL. Detection of Mycobacterium avium subspecies paratuberculosis in naturally exposed dairy heifers and associated risk factors. J Dairy Sci. 2011;94: 4669–4675. 10.3168/jds.2011-4158 21854939

[38] Whitlock RH, Widmann M, Sweeney RW, Fyock TL, Benedictus A, Mitchell RM, et al In: Nielsen S.S. (Ed.), Estimation of Parameters on the Vertical Transmission of MAP in a Low-prevalence Dairy Herd. Royal Veterinary and Agricultural University, Copenhagen, Denmark; 2005b.

[39] Whittington R, Sergeant E. Progress towards understanding the spread, detection and control of Mycobacterium avium subsp. paratuberculosis in animal populations. Australian Veterinary Journal. 2001;79: 267–278. 11349414 10.1111/j.1751-0813.2001.tb11980.x

[40] Crossley BM, Zagmutt-Vergara FJ, Fyock TL, Whitlock RH, Gardner IA. Fecal shedding of Mycobacterium avium subsp. paratuberculosis by dairy cows. Vet Microbiol. 2005;107: 257–263. 10.1016/j.vetmic.2005.01.017 15863285

[41] Sweeney RW, Collins MT, Koets AP, McGuirk SM, Roussel AJ. Paratuberculosis (Johne’s disease) in cattle and other susceptible species. J Vet Intern Med. 2012;26: 1239–1250. 10.1111/j.1939-1676.2012.01019.x 23106497

[42] Sweeney RW, Whitlock RH, Rosenberger AE. Mycobacterium paratuberculosis isolated from fetuses of infected cows not manifesting signs of the disease. Am. J. Vet. Res. 1992;53: 477–480. 1586015

[43] Aranaz A, Juan De L, Bezos J, Alvarez J, Romero B, Lozano F, et al Assessment of diagnostic tools for eradication of bovine tuberculosis in cattle co—infected with Mycobacterium bovis and M avium subsp paratuberculosis. Vet Res. 2006;37: 593–606. 10.1051/vetres:2006021 16701065

[44] Brito BP, Aly SS, Anderson RJ, Fossler CP, Garry FB, Gardner IA. Association between caudal fold tuberculin test responses and results of an ELISA for Mycobacterium avium subsp paratuberculosis and mycobacterial culture of feces in tuberculosis—free dairy herds. J Am Vet Med Assoc. 2014;244(5), 582–7. 10.2460/javma.244.5.582 . 24548233

[45] Ketterer PJ, Rogers RJ, Donald B. Pathology and tuberculin sensitivity in cattle inoculated with Mycobacterium avium complex serotypes 6, 14 and 18; 1981. Aust Vet J, 57, 61–65. 7259645 10.1111/j.1751-0813.1981.tb00445.x

[46] Köhler H, Gyra H, Zimmer K, Draqer KB, Burkert B, Lemser B, et al Immune reactions in cattle after immunization with a *Mycobacterium paratuberculosis* vaccine and implications for the diagnosis of *M paratuberculosis* and *M bovis* infections. J Vet Med B Infect Dis Vet Public Health. 2001;48: 185–195. 11393814 10.1046/j.1439-0450.2001.00443.x

[47] Dorshorst N, Collins M, Lombard J. Decision analysis model for paratuberculosis control in commercial dairy herds. Preventive Veterinary Medicine. 2006; 75: 1–2, 92–122. 10.1016/j.prevetmed.2006.02.002 16564101

[48] Groenendaal H, Wolf C. Farm-level economic analysis of the US National Johne’s Disease Demonstration Herd Project. Journal of the American Veterinary Medical Association. 2008;233: 12, 1852–58. 10.2460/javma.233.12.1852 19072597

