# Supplementary figures and images for: A nested compartmental model to assess the efficacy of paratuberculosis control measures on U.S. dairy farms
Source: PLoS One. 2018 Oct 2;13(10):e0203190. doi: 10.1371/journal.pone.0203190 (PMC6168138; doi:10.1371/journal.pone.0203190)

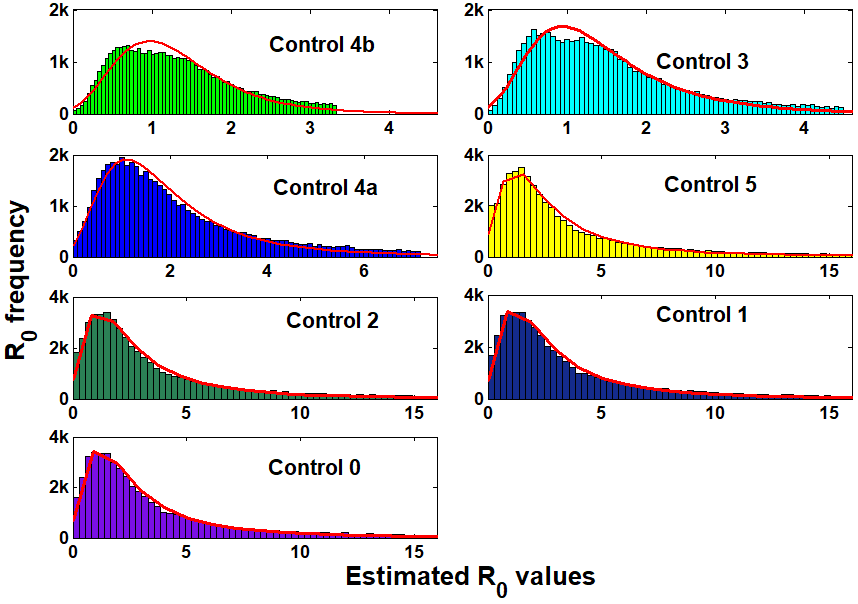

Supplement: S1 Fig — The top six panels correspond to single control measures 1–5 and the bottom panel relates to control 0 (i.e., a farm without any implemented control measure). The R0 values were calculated with 50,000 runs of the NC model. Controls 3, 4a and 4b resulted in substantially less R0 values of 1.51, 2.11, 1.31 and the calculated risks of 0.64, 0.74, and 0.60%, respectively. Note that control 4b (test and cull all the adult cows (lactating and dry) annually) is the most effective single control measure. (TIF) [file pone.0203190.s001.tif]

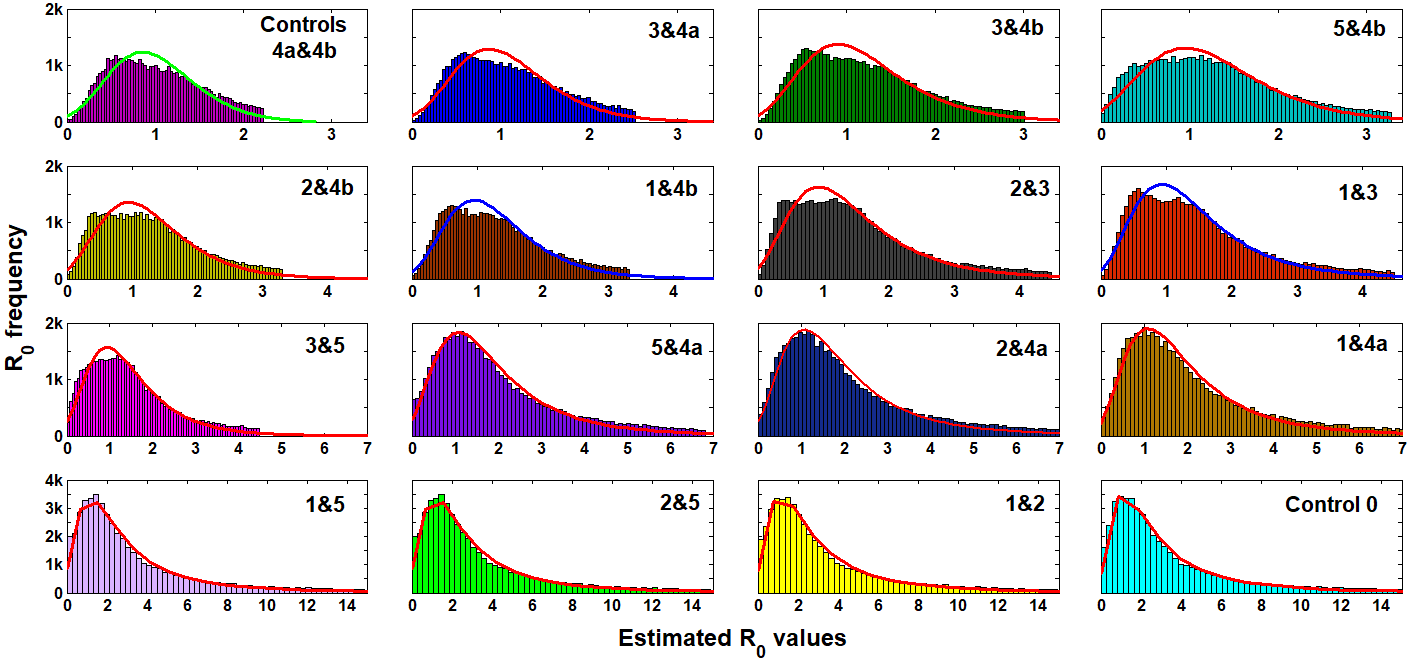

Supplement: S2 Fig — A combination of controls 4a & 4b (4a is testing at dry off on a weekly basis and culling test-positive cows; 4b is test and cull all the adult cows (lactating and dry) annually), was the most effective binary control measure with the descriptive statistics of R0 values of 1.01 and the calculated risks of 0.47%. (TIF) [file pone.0203190.s002.tif]

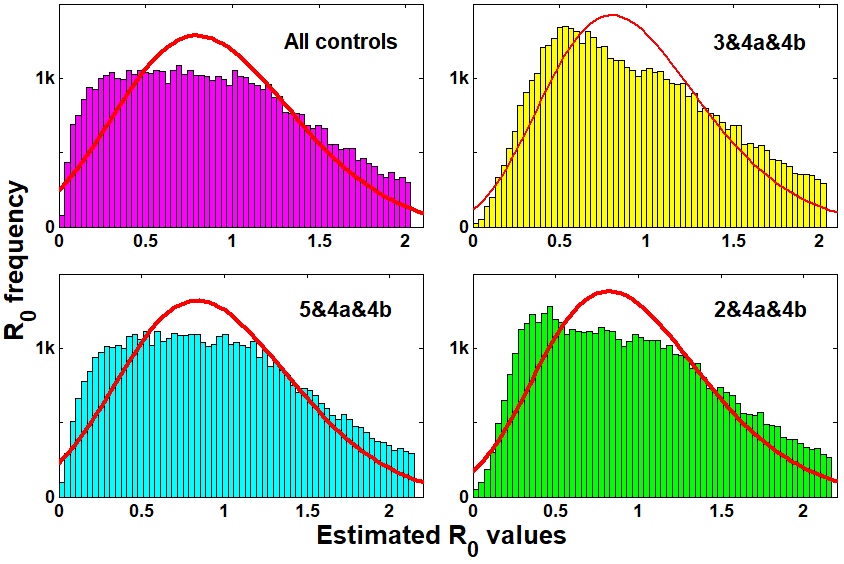

Supplement: S3 Fig — In all cases, the estimated R0 values exceed one, which indicates that the risk of infection remains greater than zero even though that all control measures have been implemented. See Table 7 for descriptive statistics of R0 values and the calculated risks. (TIF) [file pone.0203190.s003.tif]
