# Supplementary material for: A nested compartmental model to assess the efficacy of paratuberculosis control measures on U.S. dairy farms
Source: PLoS One. 2018 Oct 2;13(10):e0203190. doi: 10.1371/journal.pone.0203190 (PMC6168138; doi:10.1371/journal.pone.0203190)
Supplement: S1 Appendix — (DOCX) [file pone.0203190.s004.docx]

**S1 Appendix: *R_0_* Calculations**

**SLE Model**: *R_0_* calculation

$F=\left[ \begin{matrix} 0 & \beta_{G} \\ 0 & 0 \end{matrix} \right], V^{-1}=\left[ \begin{matrix} \frac{1}{m_{i}} & 0 \\ 0 & \frac{1}{r} \end{matrix} \right]$. And $R_{0}^{[SLE]}=\rho\left(\left[\left[\begin{matrix} 0 & \frac{\beta_{G}}{r} \\ 0 & 0 \end{matrix}\right]\right]\right)=\rho\left(\left[\begin{matrix} 0 \\ 0 \end{matrix}\right]\right)=0<1$.

Also note that the Jacobian matrix of the SLE model is given by

$$J_{DFE}=\left[\begin{matrix} -m_{i} & 0 & -\frac{b_{i}(1-\alpha)\beta_{G}}{m_{i}} \\ 0 & -m & \frac{b_{i}(1-\alpha)\beta_{G}}{m_{i}} \\ 0 & 0 & -r \end{matrix}\right]$$

Which is an upper-triangular matrix with eigenvalues: $\lambda_{1}=-r,\mathrm{and}\lambda_{2}=\lambda_{3}=-m$. Therefore, the DFE is always stable.

**SLICE Model**: *R_0_* calculation

We establish conditions for existence of the endemic equilibrium for the SLICE model with super-shedders. By setting the RHS of the SLICE model (equation (4)) equal to zero and assuming that the endemic equilibrium is given by

*Endemic equilibrium^*^ = S^*^ L^*^ I^*^ C^*^ P^*^ E^*^,* we get that

(1) $I_{j}=\frac{\sigma_{L}}{\sigma_{I}+m_{j}}L_{j}$

(2) $C_{j}=\frac{\sigma_{I}}{\sigma_{C}+m_{j}}I_{j}=\frac{\sigma_{I}\sigma_{L}}{(\sigma_{C}+m_{j})(\sigma_{I}+m_{j})}L_{j}$

(3) $P_{j}=\frac{\gamma_{I}}{r+v}I_{j}+\frac{\gamma_{C}}{r+v}C{}_{j}$

$$=\frac{\sigma_{I}}{r+v}\frac{\sigma_{L}}{\sigma_{I}+m_{j}}L_{j}+\frac{\gamma_{C}}{r+v}\frac{\sigma_{I}\sigma_{L}}{(\sigma_{C}+m_{j})(\sigma_{I}+m_{j})}L_{j}$$

$$\begin{matrix} =\frac{\sigma_{I}}{r+v}\frac{\sigma_{L}}{\sigma_{I}+m_{j}}\left(1+\frac{\gamma_{C}\sigma_{L}}{\sigma_{C}+m_{j}}\right)L_{j} \\ \end{matrix}$$

(4) $E=\frac{v}{r}\sum_{j=2}^{14} P_{j}=\frac{v}{r}\frac{\sigma_{I}}{r+v}\sum_{j=2}^{14} \left(\frac{\sigma_{L}}{\sigma_{I}+m_{j}}\left(1+\frac{\gamma_{C}\sigma_{L}}{\sigma_{C}+m_{j}}\right)\right)L_{j}$

(5) $0=b_{j}-m_{j}S_{j}-(\sigma_{L}+m_{j})L_{j}$

$$S_{j}=\frac{b_{j}}{m_{j}}-\frac{\sigma_{L}+m_{j}}{m_{j}}L_{j}$$

Using $\frac{dL_{j}}{dt}=0=\left(\frac{\beta_{I}}{N_{j}}I_{j}+\frac{\beta_{C}}{N_{j}}C_{j}+\beta_{P}P_{j}+\beta_{G}E\right)S_{j}-\left(\sigma_{L}+m_{j}\right)L_{j}$

Set $T_{j}=\frac{\beta_{I}}{N_{j}}I_{j}+\frac{\beta_{C}}{N_{j}}C_{j}+\beta_{P}P_{j}+\beta_{G}E$

**Part (i):** $\left(\frac{dL_{j}}{dt}\right):0=T_{j}S_{j}-\left(\sigma_{L}+m_{j}\right)L_{j}$

$$=L_{j}\left(\frac{T_{j}}{L_{j}}\right)\frac{1}{m_{j}}\left(b_{j}-\left(\sigma_{L}+m_{j}\right)L_{j}\right)-\left(\sigma_{L}+m_{j}\right)L_{j}$$

**Part (ii):** Now assume that $L_{j}\neq0:$ $=L_{j}\left(\frac{T_{j}}{L_{j}}\right)\frac{1}{m_{j}}\left(b_{j}-\left(\sigma_{L}+m_{j}\right)L_{j}\right)-\left(\sigma_{L}+m_{j}\right)$

Then the EE for the SLICE model with super-shedders when $L_{i}\neq0, I_{i}\neq0, C_{i}\neq0$ for *i = 6, … , 14*, and by part II we found the condition for the existence of EE is

$b_{i}{-(\sigma}_{L}+m_{i}L_{i}^{*})>0 \Longleftrightarrow R_{0}>1 and b_{1}>0$,

where $L_{i}^{*}$ is the number of Latent individuals in pen *i.*

Now we calculate the *R_0_*: we get

$F=\left[\begin{matrix} 0 & \beta_{3I} & \beta_{3C} & \beta_{3P} & \beta_{3G} \\ 0 & 0 & 0 & 0 & 0 \\ 0 & 0 & 0 & 0 & 0 \\ 0 & 0 & 0 & 0 & 0 \\ 0 & 0 & 0 & 0 & 0 \end{matrix}\right]$, $V=\left[\begin{matrix} m_{i}+\sigma_{3L} & 0 & 0 & 0 & 0 \\ -\sigma_{3L} & m_{i}+\mu_{i}+\sigma_{3I} & 0 & 0 & 0 \\ 0 & -\sigma_{3I} & m_{i}+\sigma_{3C} & 0 & 0 \\ 0 & -\gamma_{3I} & -\gamma_{3C} & r+v_{3} & 0 \\ 0 & 0 & 0 & -v_{3} & r \end{matrix}\right]$

*K = FV^-1^* =$\left[\begin{matrix} R_{0}^{[I_{i}]}+R_{0}^{[C_{i}]}+R_{0}^{[P_{i}]}+R_{0}^{[G_{i}]} & A & B & C & D \\ 0 & 0 & 0 & 0 & 0 \\ 0 & 0 & 0 & 0 & 0 \\ 0 & 0 & 0 & 0 & 0 \\ 0 & 0 & 0 & 0 & 0 \end{matrix}\right]$ , where

$R_{0}^{[I_{i}]}=\frac{\beta_{3I}\sigma_{3L}}{(m_{i}+\sigma_{3L})(m_{i}+\mu_{i}+\sigma_{3I})}$, is host specific. $R_{0}^{[C_{i}]}=\frac{\beta_{3C}\sigma_{3I}\sigma_{3L}}{(m_{i}+\sigma_{3C})(m_{i}+\sigma_{3L})(m_{i}+\mu_{i}+\sigma_{3I})}$, is super shedders specific.

$R_{0}^{[P_{i}]}=\frac{b_{i}\beta_{3P}(\gamma_{3I}m_{i}\sigma_{3L}+\gamma_{3C}\sigma_{3I}\sigma_{3L}+\gamma_{3I}\sigma_{3C}\sigma_{3L})}{(m_{i}+\sigma_{3C})(m_{i}+\sigma_{3L})(r+v_{3})(m_{i}+\mu_{i}+\sigma_{3I})}$ , is pen specific. $R_{0}^{[G_{i}]}=\frac{b_{i}v_{3}\beta_{3G}(\gamma_{3I}m_{i}\sigma_{3L}+\gamma_{3C}\sigma_{3I}\sigma_{3L}+\gamma_{3I}\sigma_{3C}\sigma_{3L})}{r(m_{i}+\sigma_{3C})(m_{i}+\sigma_{3L})(r+v_{3})(m_{i}+\mu_{i}+\sigma_{3I})}$, is general environment.

$$A=\frac{\beta_{3I}}{m_{i}+\mu_{i}+\sigma_{3I}}+\frac{\beta_{3C}\sigma_{3I}}{(m_{i}+\sigma_{3C})(m_{i}+\mu_{i}+\sigma_{3I})}+\frac{\beta_{3P}(\gamma_{3I}m_{i}+\gamma_{3C}\sigma_{3I}+\gamma_{3I}\sigma_{3C})}{(m_{i}+\sigma_{3C})(r+v_{i})(m_{i}+\mu_{i}+\sigma_{3I})}$$

$+\frac{v_{3}\beta_{3G}(\gamma_{3I}m_{i}+\gamma_{3C}\sigma_{3I}+\gamma_{3I}\sigma_{3C})}{r(m_{i}+\sigma_{3C})(r+v_{3})(m_{i}+\mu_{i}+\sigma_{3I})}$.

$B=\frac{\beta_{3C}}{m_{i}+\sigma_{3C}}+\frac{\beta_{3P}\gamma_{3C}}{(m_{i}+\sigma_{3C})(r+v_{3})}+\frac{v_{3}\beta_{3G}\gamma_{3C}}{r(m_{i}+\sigma_{3C})(r+v_{3})}$, *C = $\frac{\beta_{3P}}{r+v_{3}}+\frac{v_{3}\beta_{3G}}{r(r+v_{3})}$,* and *D = $\frac{\beta_{3G}}{r}$.*

Thus, the *R_0_* is given by

$R_{0}^{[i]}=R_{0}^{[I_{i}]}+R_{0}^{[C_{i}]}+R_{0}^{[P_{i}]}+R_{0}^{[G_{i}]}$ or

$$\begin{matrix} R_{0}^{[SLICE]}=\frac{\beta_{3I}\sigma_{3L}}{(m_{i}+\sigma_{3L})(m_{i}+\mu_{i}+\sigma_{3L})}+\frac{\beta_{3C}\sigma_{3I}\sigma_{3L}}{(m_{i}+\sigma_{3C})(m_{i}+\sigma_{3L})(m_{i}+\mu_{i}+\sigma_{3I})} \\ +\frac{b_{i}\beta_{3P}(\gamma_{3I}m_{i}\sigma_{3L}+\gamma_{3C}\sigma_{3I}\sigma_{3L}+\gamma_{3I}\sigma_{3C}\sigma_{3L})}{m_{i}(m_{i}+\sigma_{3C})(m_{i}+\sigma_{3L})(r+v_{3})(m_{i}+\mu_{i}+\sigma_{3I})}+\frac{b_{i}\beta_{3G}v_{3}(\gamma_{3I}m_{i}\sigma_{3L}+\gamma_{3C}\sigma_{3I}\sigma_{3L}+\gamma_{3I}\sigma_{3C}\sigma_{3L})}{rm_{i}(m_{i}+\sigma_{3C})(m_{i}+\sigma_{3L})(r+v_{3})(m_{i}+\mu_{i}+\sigma_{3I})}. \end{matrix}$$

$=\frac{\sigma_{3L}}{(m_{i}+\sigma_{3L})(m_{i}+\mu_{i}+\sigma_{3I})}\left(\begin{matrix} \beta_{3I}+\frac{\beta_{3C}\sigma_{3I}}{m_{i}+\sigma_{3C}}+\frac{b_{i}\beta_{3P}(\gamma_{3I}m_{i}+\gamma_{3C}\sigma_{3I}+\gamma_{3I}\sigma_{3C})}{(m_{i}+\sigma_{3C})(r+v_{3})} \\ +\frac{b_{i}v_{3}\beta_{3G}(\gamma_{3I}m_{i}+\gamma_{3C}\sigma_{3I}+\gamma_{3I}\sigma_{3C})}{r(m_{i}+\sigma_{3C})(r+v_{3})} \end{matrix}\right)$.

Hence, if *R_0_* > 1, leading to occurrence of disease and conversely, when *R_0_* < 1; the disease will die out [6, 31, 32].

**References**

6. Diekmann O, Heesterbeek JAP. Mathematical epidemiology of infectious diseases. West

Sussex, England: John Wiley & Sons; 2000.

31. Van Den Driessche P, Watmough J. Reproduction numbers and subthreshold endemic

equilibria for compartmental models of disease transmission. Math. Biosci., 2002;180:

29–48.

32. Chowell G, Brauer F. The basic reproduction number of infectious diseases: computation

and estimation using compartmental epidemic models. In: Chowell G, Hyman JM,

Bettencourt LM, Castillo-Chavez C, editors. Mathematical and statistical estimation

approaches in epidemiology. New York, NY: Springer; 2009. Pages 1–30.
