# Supplementary material for: A nested compartmental model to assess the efficacy of paratuberculosis control measures on U.S. dairy farms
Source: PLoS One. 2018 Oct 2;13(10):e0203190. doi: 10.1371/journal.pone.0203190 (PMC6168138; doi:10.1371/journal.pone.0203190)
Supplement: S2 Appendix — (DOCX) [file pone.0203190.s005.docx]

**S2 Appendix: Details of the upper limit calculation of the transmission rate in Table 3**

**^b^Details of how the upper limit of the infectious cattle transmission *β_I_* (or *β_2I_)* for heifers in pen 2 to 6 was calculated in Table 3.**

The converse of the sum of the attributable fractions for MAP detection in colostrum and teat skin surfaces linked with MAP shedding in feces of the donor cows was calculated. The number of newly infected heifers in pens 2 through 6 was estimated by dividing this converse by the sum of the attributable fractions.

Based on Pithua et al [34]: AF_tot_ = AF_1_ + AF_2_ = 18+ 20 = 38;

the converse of AF_tot_ = 100-38 = 62;

Number of newly infected heifers = 62/38 = 1.62.

Based on Aly et al [21]: AF= 34l the converse of AF = 66;
Number of newly infected heifers 66/34 =1.94.

Mean *β_I_* = (1.94+1.62)/2 = 1.78

**^c^Details of how the upper limit of the infectious cattle transmission *β_I_* (or *β_3I_)* for adult cows in pen 7 to 14 was approximated in Table 3.**

Espejo et al [30], in a 9-year longitudinal study estimated the risk of infection among adult cows introduced into MAP infected herds. Out of 17 uninfected adults that were exposed to MAP infected herds, six eventually tested positive [Risk = 6/17 = 35.29%]. The annual rate of transmission *β_I_* in uninfected adult cattle was estimated by diving the percent infected by the number of years of follow-up [35.29/9 = 3.92].

**^d^Details of how the upper limit of the general environment transmission *β_G_* (or *β_1G_)* for calves in pen 1 (hutches) was approximated in Table 3.**

The coefficient *β_G_* for transmission of MAP from the general environment to calves in pen 1 (hutches) was estimated based on data reported by Bolton et al [37]. Briefly, of the 102 calves 0-3 months of age tested by fecal culture, two were positive [2/102 = 1.9%]. The maximum annual transmission for this group of calves per year was estimated at 0.079 [~ (2/102) * 365.25/90)]. For this calculation, Herd # 4 in Bolton et al was chosen because it had the highest prevalence of MAP in calves 0-3 months of age.

^e^**Details of how the upper limit of the general environment transmission *β_G_* (or *β_2G_)* for heifers in pen 2 to 6 was approximated in Table 3.**

The coefficient *β_G_* for transmission of MAP from the general environment to heifers in pens 2 through 6 was estimated based on the total number of heifers that tested positive for MAP by fecal culture [37, Table 1, and Herds 1 to 8]. The upper range was calculated by dividing the number of test positive heifers, 3 to 24 months of age by the total number tested [~32/1266 = 0.0256]. The estimate was assumed to be the highest annual percentage of infected heifers since it spanned a range of 12 to 21 months of follow up.

**References**

21. Aly SS, Thurmond MC. [Evaluation of Mycobacterium avium subsp paratuberculosis](https://www.scopus.com/record/display.uri?eid=2-s2.0-23244449150&origin=resultslist&sort=r-f&src=s&mltEid=2-s2.0-23244449150&mltType=ref&mltAll=t&imp=t&sid=2DA202172EE6A5FB7513EB6411BE0099.wsnAw8kcdt7IPYLO0V48gA%3a640&sot=mlt&sdt=mlt&sl=512&s=REFEID%28%28%222-s2.0-0006942129%22%29+OR+%28%222-s2.0-0016651734%22%29+OR+%28%222-s2.0-0041356992%22%29+OR+%28%222-s2.0-0026556571%22%29+OR+%28%222-s2.0-0000932857%22%29+OR+%28%222-s2.0-0030184529%22%29+OR+%28%222-s2.0-0039226501%22%29+OR+%28%222-s2.0-0040756400%22%29+OR+%28%222-s2.0-0021469547%22%29+OR+%28%222-s2.0-0024970372%22%29+OR+%28%222-s2.0-2342427608%22%29+OR+%28%222-s2.0-0026089286%22%29+OR+%28%222-s2.0-0029383653%22%29+OR+%28%222-s2.0-0026161457%22%29+OR+%28%222-s2.0-0042271631%22%29+OR+%28%222-s2.0-0003663694%22%29+OR+%28%222-s2.0-1242300163%22%29+OR+%28%222-s2.0-0038044529%22%29+OR+%28%222-s2.0-0037198259%22%29%29+AND+NOT+EID+%282-s2.0-23244449150%29&recordRank=)

[infection of dairy cows attributable to infection status of the dam](https://www.scopus.com/record/display.uri?eid=2-s2.0-23244449150&origin=resultslist&sort=r-f&src=s&mltEid=2-s2.0-23244449150&mltType=ref&mltAll=t&imp=t&sid=2DA202172EE6A5FB7513EB6411BE0099.wsnAw8kcdt7IPYLO0V48gA%3a640&sot=mlt&sdt=mlt&sl=512&s=REFEID%28%28%222-s2.0-0006942129%22%29+OR+%28%222-s2.0-0016651734%22%29+OR+%28%222-s2.0-0041356992%22%29+OR+%28%222-s2.0-0026556571%22%29+OR+%28%222-s2.0-0000932857%22%29+OR+%28%222-s2.0-0030184529%22%29+OR+%28%222-s2.0-0039226501%22%29+OR+%28%222-s2.0-0040756400%22%29+OR+%28%222-s2.0-0021469547%22%29+OR+%28%222-s2.0-0024970372%22%29+OR+%28%222-s2.0-2342427608%22%29+OR+%28%222-s2.0-0026089286%22%29+OR+%28%222-s2.0-0029383653%22%29+OR+%28%222-s2.0-0026161457%22%29+OR+%28%222-s2.0-0042271631%22%29+OR+%28%222-s2.0-0003663694%22%29+OR+%28%222-s2.0-1242300163%22%29+OR+%28%222-s2.0-0038044529%22%29+OR+%28%222-s2.0-0037198259%22%29%29+AND+NOT+EID+%282-s2.0-23244449150%29&recordRank=). Journal of the American

Veterinary Medical Association. 2005;227 (3), pp. 450-454. PubMed PMID: 16121613.

30. Espejo LA, Kubat N, Godden SM, Wells S J. Eﬀect of delayed exposure of cattle to

Mycobacterium avium subsp paratuberculosis on the development of subclinical and

clinical Johne’s disease. American Journal of Veterinary Research. 2013;74(10): 1304-1310.

DOI: 10.2460/ajvr.74.10.1304

34. Pithua P, Wells SJ, Godden SM. Evaluation of the association between fecal excretion of

Mycobacterium avium subsp paratuberculosis and detection in colostrum and on teat skin

surfaces of dairy cows. Journal of the American Veterinary Medical Association.

2011;238(1): pp. 94-100.

37. Bolton MW, Pillars RB, Kaneene JB, Mauer WA, Grooms DL. Detection of Mycobacterium

avium subspecies paratuberculosis in naturally exposed dairy heifers and associated risk

factors. J Dairy Sci. 2011;94: 4669–4675. doi: 10.3168/jds.2011–4158.
