# Supplementary material for: A nested compartmental model to assess the efficacy of paratuberculosis control measures on U.S. dairy farms
Source: PLoS One. 2018 Oct 2;13(10):e0203190. doi: 10.1371/journal.pone.0203190 (PMC6168138; doi:10.1371/journal.pone.0203190)
Supplement: S3 Appendix — Supplementary Information including Summary of data collection and model formulations (Table A), R0 expressions for the NC model (pen 6–14), Estimated parameter values of the CM model (Tables B-C), Number observations and between-pen movements (Tables D-E), and Estimated sigma and mean values with 95% CI (Table F). (DOCX) [file pone.0203190.s006.docx]

**S3 Appendix**

**Summary of data collection and model formulations**

For each farm, the cattle movement data contains the following variables. Unique identifier was assigned to each cow on the farm and is designated as “uniqueid.” This designation is assigned to only one cow and is not reused when a cow dies or is sold. “ID” represents the cow's identification used on the dairy farm at the record date. Frequently dairy farms reuse the cow ID when cows die or are sold. The variable “bdate” represents the date of birth of the cow. The breed of the cow is designated as “cbrd” where H is Holstein, J is Jersey, and B is mixed breed. The date on which a cow's variables were recorded, (i.e. what pen she was in, milk produced, etc.) is termed the “record_date.” The variable “modelstate” represents the model *i* state of the cows, where *i* = 1,…, 14. The pen number is identified as “pen” and the type of pen in which the cow was housed on the record date is named “pentype.” Other data collected and summarized in Table 2 and 3 have to do with the productivity of each cow. The variable “lact” refers to the lactation or parity (the number of times that the cow has given birth). “DIM” quantifies the days in milk since calving and until dry-off. Dry-off commonly occurs 60 days prior to the next calving.

**Table A:**  A snapshot of the dairy farm data from 2011 to 2015

| **Variable** | **Farm/value** | | | | | |
| --- | --- | --- | --- | --- | --- | --- |
| **dairy** | **Farm 1** | **Farm 2** | **Farm 3** | **Farm 4** | **Farm 5** | **Farm 6** |
| uniqueid | 2485 | 25633 | 53126 | 55673 | 64018 | 68622 |
| id | 21265 | 1 | 4 | 38 | 137 | 6 |
| bdate | 1-Feb-12 | 31-May-09 | 1-Jun-03 | 10-Dec-05 | 11-Nov-04 | 19-Nov-00 |
| cbrd | J | J | H | H | H | H |
| record date | 25-Apr-14 | 10-Jan-11 | 20-Jan-11 | 15-Oct-14 | 25-Jan-11 | 23-Feb-11 |
| year | 2014 | 2011 | 2011 | 2014 | 2011 | 2011 |
| month | April | January | January | October | January | February |
| modelstate | i10 | i5 | i11 | i10 | i11 | i10 |
| pen | 12 | 56 | 9 | 8 | 6 | 1 |
| pentype | Je & Ho^1^, breeding  1^st^ lact | Springers | late lactation, milk pen | lact>4, mild  lactation | fresh cows | milk cows,  milk pen |
| lact | 1 | 0 | 3 | 6 | 4 | 8 |
| dim | 25 | 0 | 715 | 221 | 293 | 20 |
| milk | 39 | 0 | 36 | 108 | 92 | 0 |
| pctf | 3.8 | 0 | 3.3 | 3.5 | 4.2 | 0 |
| pctp | 3.1 | 0 | 3.3 | 2.8 | 3.6 | 0 |
| relv | 93 | 0 | 94 | 108 | 122 | 0 |
| sec | 35 | 0 | 878 | 7 | 119 | 0 |

^1^ Jersey and Holstein

At the time a cow is dried-off, she is not milked any more to allow her to replenish her body stores and prepare for the upcoming new lactation. “Milk” is the volume of milk produced in pounds (lbs.) on the record date; the percent of fat present in the cow's milk on that date is designated as “pctf” and the percent of protein present in the milk on that date is recorded as “pctp.” The relative value of the cow, ranked in percentile of the herd, with 100% being the average cow, is labeled “relv.” “Scc” represents the somatic cell count, a nonspecific c measure of milk quality.

**Set of ODEs for the CM model:** Following the CM model structure (see Fig. 1), the corresponding set of ordinary differential equations (ODE's) was developed as follows.

| $\begin{matrix} \frac{dN_{1}}{dt}=b_{1}+d_{5,1}N{}_{5}+d_{14,1}N_{14}-(d_{1,2}+m_{1})N_{1} \\ \frac{dN_{2}}{dt}=b_{2}+d_{1,2}N_{1}-(d_{2,3}+m_{2})N_{2} \\ \frac{dN_{3}}{dt}=b_{3}+d_{2,3}N_{2}-(d_{3,4}+m_{3})N_{3} \\ \frac{dN_{4}}{dt}=b_{4}+d_{3,4}N_{3}-(d_{4,5}+m_{4})N_{4} \\ \frac{dN_{5}}{dt}=b_{5}+d_{4,5}N_{4}-(d_{5,6}+m_{5})N_{5} \\ \frac{dN_{6}}{dt}=b_{6}+d_{5,6}N_{5}-(m_{6}+d_{6,8})N_{6} \\ \frac{dN_{7}}{dt}=b_{7}-(d_{7,10}+m_{7})N_{7}+d_{14,7}N_{14} \\ \frac{dN_{8}}{dt}=b_{8}+d_{6,8}N_{6}-(d_{8,9}+m_{8})N_{8} \\ \frac{dN_{9}}{dt}=b_{9}+d_{8,9}N_{8}-(d_{9,12}+m_{9})N_{9} \\ \frac{dN_{10}}{dt}=b_{10}+d_{7,10}N_{7}-(d_{10,11}+m_{10})N_{10} \\ \frac{dN_{11}}{dt}=b_{11}+d_{10,11}N_{10}-(d_{11,12}+m_{11})N_{11} \\ \frac{dN_{12}}{dt}=b_{12}+d_{11,12}N_{11}+d_{9,12}N_{9}-(d_{12,13}+m_{12})N_{12} \\ \frac{dN_{13}}{dt}=b_{13}+d_{12,13}N_{12}-(d_{13,14}+m_{13})N_{13} \\ \frac{dN_{14}}{dt}=b_{14}+d_{13,14}N_{13}-(d_{14,7}+m_{14})N_{14} \end{matrix}$ | (1) |
| --- | --- |

**Set of ODEs for the SLE model:** Following the SLE model structure (see Fig. 2), the corresponding set of ordinary differential equations (ODE's) was developed as follows.

| $\begin{matrix} \frac{dS_{1}}{dt}=(1-\alpha_{1})b_{1}-\beta_{1G}ES_{1}-m_{1}S_{1} \\ \frac{dL_{1}}{dt}=\alpha_{1}b_{1}+\beta_{1G}ES_{1}-m_{1}L_{1} \\ \frac{dE}{dt}=-rE \end{matrix}$ | (2) |
| --- | --- |

**Set of ODEs for the SLIE model:** Following the SLIE model structure (see Fig. 3), the corresponding set of ordinary differential equations (ODE's) was developed as follows.

| $\begin{matrix} \frac{dS_{i}}{dt}=b_{i}-\left(\frac{\beta_{2I}}{N_{i}}I_{i}+\beta_{2P}P_{i}+\beta_{2G}E\right)S_{i}-m_{i}S_{i} \\ \frac{dL_{i}}{dt}=\left(\frac{\beta_{2I}}{N_{i}}I_{i}+\beta_{2P}P_{i}+\beta_{2G}E\right)S_{i}-\left(\sigma_{2L}+m_{i}\right)L_{i} \\ \frac{dI_{i}}{dt}=\sigma_{2L}L_{i}-\left(\sigma_{2I}+\mu_{i}+m_{i}\right)I_{i} \\ \frac{dP_{i}}{dt}=\gamma_{2I}I_{i}-(r+v_{2})P_{i} \\ \frac{dE}{dt}=\sum_{i=2}^{6} v_{2}P_{i}-rE \end{matrix}$ | (3)  where *i = 2, … , 6* |
| --- | --- |

**Set of ODEs for the SLICE model:** Following the SLICE model structure (see Fig. 4), the corresponding set of ordinary differential equations (ODE's) was developed as follows.

| $\begin{matrix} \frac{dS_{i}}{dt}=b_{i}-\left(\frac{\beta_{3I}}{N_{i}}I_{i}+\frac{\beta_{3C}}{N_{i}}C_{i}+\beta_{3P}P_{i}+\beta_{3G}E\right)S_{i}-m_{i}S_{i} \\ \frac{dL_{i}}{dt}=\left(\frac{\beta_{3I}}{N_{i}}I_{i}+\frac{\beta_{3C}}{N_{i}}C_{i}+\beta_{3P}P_{i}+\beta_{3G}E\right)S_{i}-\left(\sigma_{3L}+m_{i}\right)L_{i} \\ \frac{dI_{i}}{dt}=\sigma_{3L}L_{i}-\left(\sigma_{3I}+\mu_{i}+m_{i}\right)I_{i} \\ \frac{dC_{i}}{dt}=\sigma_{3I}I_{i}-\left(\sigma_{3C}+m_{i}\right)C_{i} \\ \frac{dP_{i}}{dt}=\gamma_{3I}I_{i}+\gamma_{3C}C_{i}-(r+v_{3})P_{i} \\ \frac{dE}{dt}=\sum_{i=7}^{14} v_{3}P_{i}-rE \end{matrix}$ | (4)  where *i = 7,…,14* |
| --- | --- |

**Set of ODEs for the NC model:** Following the Nested Compartmental (NC) model structure (see Fig. 5), the corresponding set of ordinary differential equations (ODE's) was developed as follows.

***Pen 1:***

| $\begin{matrix} \frac{dS_{1}}{dt}=(1-\alpha_{1})b_{1}-\beta_{1G}ES_{1}+d_{5,1}(1-\alpha_{2})(S{}_{5}+L_{5}+I_{5})+d_{14,1}(1-\alpha_{3})(S_{14}+L_{14}+I_{14}+C_{14})-(d_{1,2}+m_{1})S_{1} \\ \frac{dL_{1}}{dt}=\alpha_{1}b_{1}+\beta_{1G}ES_{1}+\alpha_{2}d_{5,1}(S_{5}+L{}_{5}+I_{5})+\alpha_{3}d_{14,1}(S_{14}+L_{14}+I_{14}+C_{14})-(d_{1,2}+m_{1})L_{1} \end{matrix}$ | (5) |
| --- | --- |

***Pens 2 – 6:***

| $\begin{matrix} \frac{dS_{2}}{dt}=b_{2}-\left(\frac{\beta_{2I}}{N_{2}}I_{2}+\beta_{2P}P_{2}+\beta_{2G}E\right)S_{2}+d_{1,2}S_{1}-(d_{2,3}+m_{2})S_{2} \\ \frac{dL_{2}}{dt}=\left(\frac{\beta_{2I}}{N_{2}}I_{2}+\beta_{2P}P_{2}+\beta_{2G}E\right)S_{2}+d_{1,2}L_{1}-(d_{2,3}+m_{2}+\sigma_{2L})L_{2} \\ \frac{dI_{2}}{dt}=\sigma_{2L}L_{2}-(d_{2,3}+m_{2}+\mu_{2})I_{2} \\ \frac{dP_{2}}{dt}=\gamma_{2I}I_{2}-(r+\nu_{2})P_{2} \end{matrix}$ $\begin{matrix} \frac{dS_{3}}{dt}=-\left(\frac{\beta_{2I}}{N_{3}}I_{3}+\beta_{2P}P_{3}+\beta_{2G}E\right)S_{3}+d_{2,3}S_{2}-(d_{3,4}+m_{3})S_{3} \\ \frac{dL_{3}}{dt}=\left(\frac{\beta_{2I}}{N_{3}}I_{3}+\beta_{2P}P_{3}+\beta_{2G}E\right)S_{3}+d_{2,3}L_{2}-(d_{3,4}+m_{3}+\sigma_{2L})L_{3} \\ \frac{dI_{3}}{dt}=\sigma_{2L}L_{3}+d_{2,3}I_{2}-(d_{3,4}+m_{3}+\mu_{3})I_{3} \\ \frac{dP_{3}}{dt}=\gamma_{2I}I_{3}-(r+\nu_{2})P_{3} \end{matrix}$ $\begin{matrix} \frac{dS_{4}}{dt}=-\left(\frac{\beta_{2I}}{N_{4}}I_{4}+\beta_{2P}P_{4}+\beta_{2G}E\right)S_{4}+d_{3,4}S_{3}-(d_{4,5}+m_{4})S_{4} \\ \frac{dL_{4}}{dt}=\left(\frac{\beta_{2I}}{N_{4}}I_{4}+\beta_{2P}P_{4}+\beta_{2G}E\right)S_{4}+d_{3,4}L_{3}-(d_{4,5}+m_{4}+\sigma_{2L})L_{4} \\ \frac{dI_{4}}{dt}=\sigma_{2L}L_{4}+d_{3,4}I_{3}-(d_{4,5}+m_{4}+\mu_{4})I_{4} \\ \frac{dP_{4}}{dt}=\gamma_{2I}I_{4}-(r+\nu_{2})P_{4} \end{matrix}$ $\frac{dS_{5}}{dt}=b_{5}-\left(\frac{\beta_{2I}}{N_{5}}I_{5}+\beta_{2P}P_{5}+\beta_{2G}E\right)S_{5}+d_{4,5}S_{4}-(d_{5,6}+m_{5})S_{5}$ $\begin{matrix} \frac{dL_{5}}{dt}=\left(\frac{\beta_{2I}}{N_{5}}I_{5}+\beta_{2P}P_{5}+\beta_{2G}E\right)S_{5}+d_{4,5}L_{4}-(d_{5,6}+m_{5}+\sigma_{2L})L_{5} \\ \frac{dI_{5}}{dt}=\sigma_{2L}L_{5}+d_{4,5}I_{4}-(d_{5,6}+m_{5}+\mu_{5})I_{5} \\ \frac{dP_{5}}{dt}=\gamma_{2I}I_{5}-(r+\nu_{2})P_{5} \end{matrix}$ $\begin{matrix} \frac{dS_{6}}{dt}=b_{6}-\left(\frac{\beta_{2I}}{N_{6}}I_{6}+\beta_{2P}P_{6}+\beta_{2G}E\right)S_{6}+d_{5,6}S_{5}-(d_{6,8}+m_{6})S_{6} \\ \frac{dL_{6}}{dt}=\left(\frac{\beta_{2I}}{N_{6}}I_{6}+\beta_{2P}P_{6}+\beta_{2G}E\right)S_{6}+d_{5,6}L_{5}-(d_{6,8}+m_{6}+\sigma_{2L})L_{6} \\ \frac{dI_{6}}{dt}=\sigma_{2L}L_{6}+d_{5,6}I_{5}-(d_{6,8}+m_{6}+\mu_{6})I_{6} \\ \frac{dP_{6}}{dt}=\gamma_{2I}I_{6}-(r+\nu_{2})P_{6} \end{matrix}$ | (6)    (6) |
| --- | --- |

***Pens 7 – 14:***

| $\begin{matrix} \frac{dS_{7}}{dt}=b_{7}-\left(\frac{\beta_{3I}}{N_{7}}I_{7}+\frac{\beta_{3C}}{N_{7}}C_{7}+\beta_{3P}P_{7}+\beta_{3G}E\right)S_{7}+d_{14,7}S_{14}-(d_{7,10}+m_{7})S_{7} \\ \frac{dL_{7}}{dt}=\left(\frac{\beta_{3I}}{N_{7}}I_{7}+\frac{\beta_{3C}}{N_{7}}C_{7}+\beta_{3P}P_{7}+\beta_{3G}E\right)S_{7}+d_{14,7}L_{14}-\left(\sigma_{3L}+d_{7,10}+m_{7}\right)L_{7} \\ \frac{dI_{7}}{dt}=\sigma_{3L}L_{7}+d_{14,7}I_{14}-\left(d_{7,10}+m_{7}+\mu_{7}+\sigma_{3I}\right)I_{7} \\ \frac{dC_{7}}{dt}=\sigma_{3I}I_{7}+d_{14,7}C_{14}-\left(\sigma_{3C}+d_{7,10}+m_{7}\right)C_{7} \\ \frac{dP_{7}}{dt}=\gamma_{3I}I_{7}+\gamma_{3C}C_{7}-(r+v_{3})P_{7} \end{matrix}$ $\begin{matrix} \frac{dS_{8}}{dt}=b_{8}-\left(\frac{\beta_{3I}}{N_{8}}I_{8}+\frac{\beta_{3C}}{N_{8}}C_{8}+\beta_{3P}P_{8}+\beta_{3G}E\right)S_{8}+d_{6,8}S_{6}-(d_{8,9}+m_{8})S_{8} \\ \frac{dL_{8}}{dt}=\left(\frac{\beta_{3I}}{N_{8}}I_{8}+\frac{\beta_{3C}}{N_{8}}C_{8}+\beta_{3P}P_{8}+\beta_{3G}E\right)S_{8}+d_{6,8}L_{6}-\left(\sigma_{3L}+d_{8,9}+m_{8}\right)L_{8} \\ \frac{dI_{8}}{dt}=\sigma_{3L}L_{8}+d_{6,8}I_{6}-\left(\sigma_{3I}+d_{8,9}+m_{8}+\mu_{8}\right)I_{8} \end{matrix}$ $\frac{dC_{8}}{dt}=\sigma_{3I}I_{8}+d_{6,8}C_{6}-\left(\sigma_{3C}+d_{8,9}+m_{8}\right)C_{8}$ $\frac{dP_{8}}{dt}=\gamma_{3I}I_{8}+\gamma_{3C}C_{8}-(r+v_{3})P_{8}$ $\begin{matrix} \frac{dS_{9}}{dt}=b_{9}-\left(\frac{\beta_{3I}}{N_{9}}I_{9}+\frac{\beta_{3C}}{N_{9}}C_{9}+\beta_{3P}P_{9}+\beta_{3G}E\right)S_{9}+d_{8,9}S_{8}-(d_{9,12}+m_{9})S_{9} \\ \frac{dL_{9}}{dt}=\left(\frac{\beta_{3I}}{N_{9}}I_{9}+\frac{\beta_{3C}}{N_{9}}C_{9}+\beta_{3P}P_{9}+\beta_{3G}E\right)S_{9}+d_{8,9}L_{8}-\left(\sigma_{3L}+d_{9,12}+m_{9}\right)L_{9} \\ \frac{dI_{9}}{dt}=\sigma_{3L}L_{9}+d_{8,9}I_{8}-\left(\sigma_{3I}+d_{9,12}+m_{9}+\mu_{9}\right)I_{9} \\ \frac{dC_{9}}{dt}=\sigma_{3I}I_{9}+d_{8,9}C_{8}-\left(\sigma_{3C}+d_{9,12}+m_{9}\right)C_{9} \\ \frac{dP_{9}}{dt}=\gamma_{3I}I_{9}+\gamma_{3C}C_{9}-(r+v_{3})P_{9} \end{matrix}$ $\begin{matrix} \frac{dS_{10}}{dt}=b_{10}-\left(\frac{\beta_{3I}}{N_{10}}I_{10}+\frac{\beta_{3C}}{N_{10}}C_{10}+\beta_{3P}P_{10}+\beta_{3G}E\right)S_{10}+d_{7,10}S_{7}-(d_{10,11}+m_{10})S_{10} \\ \frac{dL_{10}}{dt}=\left(\frac{\beta_{3I}}{N_{10}}I_{10}+\frac{\beta_{3C}}{N_{10}}C_{10}+\beta_{3P}P_{10}+\beta_{3G}E\right)S_{10}+d_{7,10}L_{7}-\left(\sigma_{3L}+d_{10,11}+m_{10}\right)L_{10} \\ \frac{dI_{10}}{dt}=\sigma_{3L}L_{10}+d_{7,10}I_{7}-\left(\sigma_{3I}+d_{10,11}+m_{10}+\mu_{10}\right)I_{10} \\ \frac{dC_{10}}{dt}=\sigma_{3I}I_{10}+d_{7,10}C_{7}-\left(\sigma_{3C}+d_{10,11}+m_{10}\right)C_{10} \\ \frac{dP_{10}}{dt}=\gamma_{3I}I_{10}+\gamma_{3C}C_{10}-(r+v_{3})P_{10} \end{matrix}$ $\begin{matrix} \frac{dS_{11}}{dt}=b_{11}-\left(\frac{\beta_{3I}}{N_{11}}I_{11}+\frac{\beta_{3C}}{N_{11}}C_{11}+\beta_{3P}P_{11}+\beta_{3G}E\right)S_{11}+d_{10,11}S_{10}-(d_{11,12}+m_{11})S_{11} \\ \frac{dL_{11}}{dt}=\left(\frac{\beta_{3I}}{N_{11}}I_{11}+\frac{\beta_{3C}}{N_{11}}C_{11}+\beta_{3P}P_{11}+\beta_{3G}E\right)S_{11}+d_{10,11}L_{10}-\left(\sigma_{3L}+d_{11,12}+m_{11}\right)L_{11} \\ \frac{dI_{11}}{dt}=\sigma_{3L}L_{11}+d_{10,11}I_{10}-\left(\sigma_{3I}+d_{11,12}+m_{11}+\mu_{11}\right)I_{11} \\ \frac{dC_{11}}{dt}=\sigma_{3I}I_{11}+d_{10,11}C_{10}-\left(\sigma_{3C}+d_{11,12}+m_{11}\right)C_{11} \\ \frac{dP_{11}}{dt}=\gamma_{3I}I_{11}+\gamma_{3C}C_{11}-(r+v_{3})P_{11} \end{matrix}$ $\begin{matrix} \frac{dS_{12}}{dt}=b_{12}-\left(\frac{\beta_{3I}}{N_{12}}I_{12}+\frac{\beta_{3C}}{N_{12}}C_{12}+\beta_{3P}P_{12}+\beta_{3G}E\right)S_{12}+d_{11,12}S_{11}+d_{9,12}S_{9}-(d_{12,13}+m_{12})S_{12} \\ \frac{dL_{12}}{dt}=\left(\frac{\beta_{3I}}{N_{12}}I_{12}+\frac{\beta_{3C}}{N_{12}}C_{12}+\beta_{3P}P_{12}+\beta_{3G}E\right)S_{12}+d_{11,12}L_{11}+d_{9,12}L_{9}-\left(\sigma_{3L}+d_{12,13}+m_{12}\right) \end{matrix}$ $\frac{dI_{12}}{dt}=\sigma_{3L}L_{12}+d_{11,12}I_{11}+d_{9,12}I_{9}-\left(\sigma_{3I}+d_{12,13}+m_{12}+\mu_{12}\right)I_{12}$ $\frac{dC_{12}}{dt}=\sigma_{3I}I_{12}+d_{11,12}C_{11}+d_{9,12}C_{9}-\left(\sigma_{3C}+d_{12,13}+m_{12}\right)C_{12}$ $\begin{matrix} \frac{dP_{12}}{dt}=\gamma_{3I}I_{12}+\gamma_{3C}C_{12}-(r+v_{3})P_{12} \\ \frac{dS_{13}}{dt}=b_{13}-\left(\frac{\beta_{3I}}{N_{13}}I_{13}+\frac{\beta_{3C}}{N_{13}}C_{13}+\beta_{3P}P_{13}+\beta_{3G}E\right)S_{13}+d_{12,13}S_{12}-(d_{13,14}+m_{13})S_{13} \\ \frac{dL_{13}}{dt}=\left(\frac{\beta_{3I}}{N_{13}}I_{13}+\frac{\beta_{3C}}{N_{13}}C_{13}+\beta_{3P}P_{13}+\beta_{3G}E\right)S_{13}+d_{12,13}L_{12}-\left(\sigma_{3L}+d_{13,14}+m_{13}\right)L_{13} \\ \frac{dI_{13}}{dt}=\sigma_{3L}L_{13}+d_{12,13}I_{12}-\left(\sigma_{3I}+d_{13,14}+m_{13}+\mu_{13}\right)I_{13} \\ \frac{dC_{13}}{dt}=\sigma_{3I}I_{13}+d_{12,13}C_{12}-\left(\sigma_{3C}+d_{13,14}+m_{13}\right)C_{13} \\ \frac{dP_{13}}{dt}=\gamma_{3I}I_{13}+\gamma_{3C}C_{13}-(r+v_{3})P_{13} \\ \frac{dS_{14}}{dt}=b_{14}-\left(\frac{\beta_{3I}}{N_{14}}I_{14}+\frac{\beta_{3C}}{N_{14}}C_{14}+\beta_{3P}P_{14}+\beta_{3G}E\right)S_{14}+d_{13,14}S_{13}-(d_{14,7}+m_{14})S_{14} \\ \frac{dL_{14}}{dt}=\left(\frac{\beta_{3I}}{N_{14}}I_{14}+\frac{\beta_{3C}}{N_{14}}C_{14}+\beta_{3P}P_{14}+\beta_{3G}E\right)S_{14}+d_{13,14}L_{13}-\left(\sigma_{3L}+d_{14,7}+m_{14}\right)L_{14} \\ \frac{dI_{14}}{dt}=\sigma_{3L}L_{14}+d_{13,14}I_{13}-\left(\sigma_{3I}+d_{14,7}+m_{14}+\mu_{14}\right)I_{14} \\ \frac{dC_{14}}{dt}=\sigma_{3I}I_{14}+d_{13,14}C_{13}-\left(\sigma_{3C}+d_{14,7}+m_{14}\right)C_{14} \\ \frac{dP_{14}}{dt}=\gamma_{3I}I_{14}+\gamma_{3C}C_{14}-(r+v_{3})P_{14} \end{matrix}$ | (7)  (7)  (7) |
| --- | --- |

| $\begin{matrix} \frac{dE}{dt}=\sum_{i=1}^{1} v_{1}P_{i}-rE \\ \frac{dE}{dt}=\sum_{i=2}^{6} v_{2}P_{i}-rE \\ \frac{dE}{dt}=\sum_{i=7}^{14} v_{3}P_{i}-rE \end{matrix}$ | (8) |
| --- | --- |

Each of the rates *m_i_, b_i_,* for *i* = 1, . . ., 14 represent the mean culling and all-cause mortality rate, and purchase and birth rate, respectively. Each of the coefficients *β_I_, β_C_, β_P_*, and *β_G_* represent the transmission rate due to infectious cattle (*β_I_*), super-shedder cattle (*β_C_*), pen environment (*β_P_*), and general environment due to recycled lagoon water used to flush the entire dairy (*β_G_*). Each of *σ_L_, σ_I_, σ_C_, ϒ_I_, ϒ_C_, v,* $\mu$,and *r* represent infection state rates for change from the latent stage (*σ_L_*), infectious (*σ_I_*), super shedder (*σ_C_*), mean infectious shedding (*ϒ_I_*), mean super-shedder shedding (*ϒ_C_*), transition rate from a pen to the general environment (*v*), farm animal removal rate ($\mu$), and the duration of pathogen survival (*r*).

***R_0_* expressions for the NC model (pen 6 – 14)**

For the nested compartmental model, the *R_0_* expression for pen 6 to 14 are listed as follows:

$$R_{0}^{[6]}=\frac{\beta_{2I}\sigma_{2L}}{(d_{6,8}+m_{6}+\mu_{6})(d_{6,8}+m_{8}+\sigma_{2L})}+\frac{\beta_{2P}\gamma_{2I}\sigma_{2L}(b_{6}+S_{5}d_{5,6})}{(d_{6,8}+m_{6})(r+v_{2})(d_{6,8}+m_{6}+\mu_{6})(d_{6,8}+m_{6}+\sigma_{2L})}$$

$+\frac{\beta_{2G}\gamma_{2I}\sigma_{2L}v_{2}(b_{6}+S_{5}d_{5,6})}{r(d_{6,8}+m_{6})(r+v_{2})(d_{6,8}+m_{6}+\mu_{6})(d_{6,8}+m_{6}+\sigma_{2L})}$.

$$R_{0}^{[7]}=\frac{\beta_{3I}\sigma_{3L}}{(d_{7,10}+m_{7}+\sigma_{3L})(d_{7,10}+m_{7}+\mu_{7}+\sigma_{3I})}$$

$$+\frac{\beta_{3C}\sigma_{3I}\sigma_{3L}}{(d_{7,10}+m_{7}+\sigma_{3C})(d_{7,10}+m_{7}+\sigma_{3L})(d_{7,10}+m_{7}+\mu_{7}+\sigma_{3I})}$$

$$+\frac{\beta_{3P}\sigma_{3L}(b_{7}+S_{14}d_{14,7})(d_{7,10}\gamma_{3I}+\gamma_{3I}m_{7}+\gamma_{3C}\sigma_{3I}+\gamma_{3I}\sigma_{3C})}{(d_{7,10}+m_{7})(r+v_{3})(d_{7,10}+m_{7}+\sigma_{3C})(d_{7,10}+m_{7}+\sigma_{3L})(d_{7,10}+m_{7}+\mu_{7}+\sigma_{3I})}$$

$+\frac{\beta_{3G}\sigma_{3L}v_{3}(b_{7}+S_{14}d_{14,7})(d_{7,10}\gamma_{3I}+\gamma_{3I}m_{7}+\gamma_{3C}\sigma_{3I}+\gamma_{3I}\sigma_{3C}}{r(d_{7,10}+m_{7})(r+v_{3})(d_{7,10}+m_{7}+\sigma_{3C})(d_{7,10}+m_{7}+\sigma_{3L})(d_{7,10}+m_{7}+\mu_{7}+\sigma_{3I})}$.

$$R_{0}^{[8]}=\frac{\beta_{3I}\sigma_{3L}}{(d_{8,9}+m_{8}+\sigma_{3L})(d_{8,9}+m_{8}+\mu_{8}+\sigma_{3I})}+\frac{\beta_{3C}\sigma_{3I}\sigma_{3L}}{(d_{8,9}+m_{8}+\sigma_{3C})(d_{8,9}+m_{8}+\sigma_{3L})(d_{8,9}+m_{8}+\mu_{8}+\sigma_{3I})}$$

$$+\frac{\beta_{3P}\sigma_{3L}(b_{8}+S_{6}d_{6,8})(d_{8,9}\gamma_{3I}+\gamma_{3I}m_{8}+\gamma_{3C}\sigma_{3I}+\gamma_{3I}\sigma_{3C})}{(d_{8,9}+m_{8})(r+v_{3})(d_{8,9}+m_{8}+\sigma_{3C})(d_{8,9}+m_{8}+\sigma_{3L})(d_{8,9}+m_{8}+\mu_{8}+\sigma_{3I})}$$

$+\frac{\beta_{3G}\sigma_{3L}v_{3}(b_{9}+S_{8}d_{8,9})(d_{9,12}\gamma_{3I}+\gamma_{3I}m_{9}+\gamma_{3C}\sigma_{3I}+\gamma_{3I}\sigma_{3C})}{r(d_{8,9}+m_{8})(r+v_{3})(d_{8,9}+m_{8}+\sigma_{3C})(d_{8,9}+m_{8}+\sigma_{3L})(d_{8,9}+m_{8}+\mu_{8}+\sigma_{3I})}$.

$$R_{0}^{[9]}=\frac{\beta_{3I}\sigma_{3L}}{(d_{9,12}+m_{9}+\sigma_{3L})(d_{9,12}+m_{9}+\mu_{9}+\sigma_{3I})}$$

$$+\frac{\beta_{3C}\sigma_{3I}\sigma_{3L}}{(d_{9,12}+m_{9}+\sigma_{3C})(d_{9,12}+m_{9}+\sigma_{3L})(d_{9,12}+m_{9}+\mu_{9}+\sigma_{3I})}$$

$$+\frac{\beta_{3P}\sigma_{3L}(b_{9}+S_{8}d_{8,9})(d_{9,12}\gamma_{3I}+\gamma_{3I}m_{9}+\gamma_{3C}\sigma_{3I}+\gamma_{3I}\sigma_{3C})}{(d_{9,12}+m_{9})(r+v_{3})(d_{9,12}+m_{9}+\sigma_{3C})(d_{9,12}+m_{9}+\sigma_{3L})(d_{9,12}+m_{9}+\mu_{9}+\sigma_{3I})}$$

$+\frac{\beta_{3G}\sigma_{3L}v_{3}(b_{9}+S_{8}d_{8,9})(d_{9,12}\gamma_{3I}+\gamma_{3I}m_{9}+\gamma_{3C}\sigma_{3I}+\gamma_{3I}\sigma_{3C})}{r(d_{9,12}+m_{9})(r+v_{3})(d_{9,12}+m_{9}+\sigma_{3C})(d_{9,12}+m_{9}+\sigma_{3L})(d_{9,12}+m_{9}+\mu_{9}+\sigma_{3I})}$.

$$R_{0}^{[10]}=\frac{\beta_{3I}\sigma_{3L}}{(d_{10,11}+m_{10}+\sigma_{3L})(d_{10,11}+m_{10}+\mu_{10}+\sigma_{3I})}$$

$$+\frac{\beta_{3C}\sigma_{3I}\sigma_{3L}}{(d_{10,11}+m_{10}+\sigma_{3C})(d_{10,11}+m_{10}+\sigma_{3L})(d_{10,11}+m_{10}+\mu_{10}+\sigma_{3I})}$$

$$+\frac{\beta_{3P}\sigma_{3L}(b_{10}+S_{7}d_{7,10})(d_{10,11}\gamma_{3I}+\gamma_{3I}m_{10}+\gamma_{3C}\sigma_{3I}+\gamma_{3I}\sigma_{3C})}{(d_{10,11}+m_{10})(r+v_{3})(d_{10,11}+m_{10}+\sigma_{3C})(d_{10,11}+m_{10}+\sigma_{3L})(d_{10,11}+m_{10}+\mu_{10}+\sigma_{3I})}$$

$+\frac{\beta_{3G}\sigma_{3L}v_{3}(b_{10}+S_{7}d_{7,10})(d_{10,11}\gamma_{3I}+\gamma_{3I}m_{10}+\gamma_{3C}\sigma_{3I}+\gamma_{3I}\sigma_{3C})}{r(d_{10,11}+m_{10})(r+v_{3})(d_{10,11}+m_{10}+\sigma_{3C})(d_{10,11}+m_{10}+\sigma_{3L})(d_{10,11}+m_{10}+\mu_{10}+\sigma_{3I})}$.

$R_{0}^{[11,13]}=\frac{\beta_{3I}\sigma_{3L}}{(d_{i,j+1}+m_{i}+\mu_{i})(d_{i,j+1}+m_{i}+\sigma_{3L})}+\frac{\beta_{3P}\gamma_{3I}\sigma_{3L}(b_{i}+S_{i-1}d_{i-1,j})}{(d_{i,j+1}+m_{i})(r+v_{3})(d_{i,j+1}+m_{i}+\mu_{i})(d_{i,j+1}+m_{i}+\sigma_{3L})}$

$+\frac{\beta_{3G}\gamma_{3I}\sigma_{3L}v_{3}(b_{i}+S_{i-1}d_{i,j})}{r(d_{i,j+1}+m_{i})(r+v_{3})(d_{i,j+1}+m_{i}+\mu_{i})(d_{i,j+1}+m_{i}+\sigma_{3L})}$**,** where *i, j =11 and 13.*

$$R_{0}^{[12]}=\frac{\beta_{3I}\sigma_{3L}}{(d_{12,13}+m_{12}+\sigma_{3L})(d_{12,13}+m_{12}+\mu_{12}+\sigma_{3I})}$$

$$+\frac{\beta_{3C}\sigma_{3I}\sigma_{3L}}{(d_{12,13}+m_{12}+\sigma_{3C})(d_{12,13}+m_{13}+\sigma_{3L})(d_{12,13}+m_{12}+\mu_{12}+\sigma_{3I})}$$

$$+\frac{\beta_{3P}\sigma_{3L}(b_{12}+S_{11}d_{9,12}+S_{11}d_{11,12})(d_{12,13}\gamma_{3I}+\gamma_{3I}m_{12}+\gamma_{3C}\sigma_{3I}+\gamma_{3I}\sigma_{3C})}{(d_{12,13}+m_{12})(r+v_{3})(d_{12,13}+m_{12}+\sigma_{3C})(d_{12,13}+m_{12}+\sigma_{3L})(d_{12,13}+m_{13}+\mu_{12}+\sigma_{3I})}$$

$+\frac{\beta_{3G}\sigma_{3L}v_{3}(b_{12}+S_{9}d_{9,12}+S_{11}d_{11,12})(d_{12,13}\gamma_{3I}+\gamma_{3I}m_{12}+\gamma_{3C}\sigma_{3I}+\gamma_{3I}\sigma_{3C})}{r(d_{12,13}+m_{12})(r+v_{3})(d_{12,13}+m_{12}+\sigma_{3C})(d_{12,13}+m_{12}+\sigma_{3L})(d_{12,13}+m_{12}+\mu_{12}+\sigma_{3I})}$.

$$R_{0}^{[14]}=\frac{\beta_{3I}\sigma_{3L}}{(d_{14,7}+m_{14}+\sigma_{3L})(d_{14,7}+m_{14}+\mu_{14}+\sigma_{3I})}$$

$$+\frac{\beta_{3C}\sigma_{3I}\sigma_{3L}}{(d_{14,7}+m_{14}+\sigma_{3C})(d_{14,7}+m_{14}+\sigma_{3L})(d_{14,7}+m_{14}+\mu_{14}+\sigma_{3I})}$$

$$+\frac{\beta_{3P}\sigma_{3L}(b_{14}+S_{13}d_{13,14})(d_{14,7}\gamma_{3I}+\gamma_{3I}m_{14}+\gamma_{3C}\sigma_{3I}+\gamma_{3I}\sigma_{3C})}{(d_{14,7}+m_{14})(r+v_{3})(d_{14,7}+m_{14}+\sigma_{3C})(d_{14,7}+m_{14}+\sigma_{3L})(d_{14,7}+m_{14}+\mu_{14}+\sigma_{3I})}$$

$$+\frac{\beta_{3G}\sigma_{3L}v_{3}(b_{14}+S_{14}d_{13,14})(d_{14,7}\gamma_{3I}+\gamma_{3I}m_{14}+\gamma_{3C}\sigma_{3I}+\gamma_{3I}\sigma_{3C})}{r(d_{14,7}+m_{14})(r+v_{3})(d_{14,7}+m_{14}+\sigma_{3C})(d_{14,7}+m_{14}+\sigma_{3L})(d_{14,7}+m_{14}+\mu_{14}+\sigma_{3I})}$$

Thus, if $R_{0}^{[\mathrm{i}]}>1$for *i = 2, . . . , 14*, leading to occurrence of disease. However, if $R_{0}^{[\mathrm{i}]}<1$ for all *i* = 2, …, 14 does not guarantee that the disease will die out. If we let *d_i,j_* = 0 for all pens where *d_i,j_* is the rate of moving cattle from pen *i* to pen *j*, and *i, j* = 1, …, 14. Then all the *R_0_* expressions above simplify such that $R_{0}^{[1]},R_{0}^{[2,...,6]},$and $R_{0}^{[7,...,14]}$correspond to SLE, SLIE, and SLICE models, respectively.

**Estimated parameter values of the CM model**

The following two tables provide the details of the estimated rates of the CM model. This was done using the Matlab optimization toolbox, file fmincon.m

**Table B:** Estimated rates of moving cows between pens (*i* to *j*) on 4 dairy farms based on dairy herd improvement backups over a 5-year period.

| **Parameter^a^**  **(pen i, pen j)** | **Farm 1a** | **Farm 1b** | **Farm 2** | **Farm 3** | **Farm 4** | **Mean rate** |
| --- | --- | --- | --- | --- | --- | --- |
| d_1,2_ | 5.75 | 3.45 | 6.00 | 6.00 | 3.00 | 4.84 |
| d_2,3_ | 1.14 | 1.74 | 1.99 | 1.99 | 1.00 | 1.57 |
| d_3,4_ | 11.89 | 11.61 | 11.99 | 4.00 | 4.00 | 8.70 |
| d_4,5_ | 1.70 | 1.69 | 1.53 | 1.50 | 1.50 | 1.58 |
| d_5,1_ | 233.80 | 54.42 | 52.08 | 52.08 | 52.08 | 88.89 |
| d_5,6_ | 8.15 | 8.28 | 8.67 | 8.66 | 8.66 | 8.48 |
| d_6,8_ | 193.83 | 205.49 | 17.39 | 191.25 | 191.24 | 159.84 |
| d_7,10_ | 209.01 | 19.79 | 17.39 | 159.79 | 159.79 | 113.15 |
| d_8,9_ | 6.12 | 4.52 | 52.08 | 3.04 | 3.04 | 13.76 |
| d_9,12_ | 1.27 | 1.28 | 1.50 | 1.49 | 1.50 | 1.41 |
| d_10,11_ | 51.67 | 8.34 | 3.03 | 33.51 | 33.51 | 26.01 |
| d_11,12_ | 1.23 | 1.38 | 1.00 | 1.50 | 1.49 | 1.32 |
| d_12,13_ | 11.01 | 11.43 | 10.72 | 6.65 | 6.65 | 9.29 |
| d_13,14_ | 97.48 | 342.60 | 90.94 | 225.49 | 225.49 | 196.40 |
| d_14,1_ | 195.18 | 165.01 | 52.08 | 52.08 | 52.08 | 103.29 |
| d_14,7_ | 226.02 | 220.73 | 370.37 | 370.37 | 370.37 | 311.57 |

^a^The parameter *d_i,j_* is the mean monthly rate of cattle movement from pen *i* to pen *j*, where

*i, j = 1, . . . , 14*

**Table C:** Estimated parameter values using the data of all farms. The unit for *m_i_* and *b_i_* are 1/year, cows/year for *i* = 1,…, 14, respectively.

| **Birth/**  **buying** | **Average** | **Range used** | **Natural death/Culling** | **Average** | **Range used** | **Pen** |
| --- | --- | --- | --- | --- | --- | --- |
| b_1_ | 0 | 0 – 0 | m_1_ | 3.04 | 0 – 5 | 1 |
| b_2_ | 0.00006 | 0 – 0.003 | m_2_ | 1.90 | 0.99 – 5.49 | 2 |
| b_3_ | 0.12 | 0 – 0.29 | m_3_ | 2.78 | 0 – 10 | 3 |
| b_4_ | 0.00001 | 0 – 0.00005 | m_4_ | 2.03 | 0.81 – 3 | 4 |
| b_5_ | 0 | 0 – 0 | m_5_ | 0.21 | 0 – 1 | 5 |
| b_6_ | 0 | 0 – 0 | m_6_ | 0.04 | 0 – 1 | 6 |
| b_7_ | 0.022 | 0 – 0.1 | m_7_ | 3.14 | 0.07 – 5 | 7 |
| b_8_ | 0.024 | 0 – 0.12 | m_8_ | 1.05 | 0.23 – 2 | 8 |
| b_9_ | 0 | 0 – 0 | m_9_ | 1.22 | 0.11 – 2 | 9 |
| b_10_ | 0.002 | 0 – 0.01 | m_10_ | 1.28 | 0.45 – 2 | 10 |
| b_11_ | 0.0008 | 0 – 0.0039 | m_11_ | 1.60 | 0.02 – 2 | 11 |
| b_12_ | 0.008 | 0 – 0.04 | m_12_ | 1.01 | 0.02 – 2 | 12 |
| b_13_ | 0.056 | 0 – 0.28 | m_13_ | 2.85 | 0.24 – 6 | 13 |
| b_14_ | 0 | 0 – 0 | m_14_ | 1.78 | 1 – 3.35 | 14 |

Pens 3 and 11 had much higher entry rate (i.e. purchasing rate). Pens 3, 4 and 11 had much higher mortality. Pen 6 had the lowest mortality rate. Pens 3 and 11 had much higher entry rate (i.e. purchasing rate)

**Number observations and between-pen movements**

**Table D:** Total number of observations recorded from each farm over a 5-year period

| **Farm** | **2011** | **2012** | **2013** | **2014** | **2015** | **Total^*^** |
| --- | --- | --- | --- | --- | --- | --- |
| Farm 1 | 34 | 4 | 0 | 49 | 8 | 95 |
| Farm 2 | 12 | 12 | 14 | 22 | 9 | 69 |
| Farm 3 | 24 | 18 | 30 | 39 | 10 | 121 |
| Farm 4 | 70 | 34 | 57 | 120 | 33 | 314 |
| all | 12 | 12 | 14 | 22 | 9 | 69 |

^a^No reading days recorded. ^*^Total number of backups

**Table E:** Number of between-Pen movements for cattle on 4 dairy farms

| **Farm** | **2011** | **2012** | **2013** | **2014** | **2015** | **Total** |
| --- | --- | --- | --- | --- | --- | --- |
| Farm 1 | 314,425 | 32,760 | x | 447,335 | 74,600 | 869,120 |
| Farm 2 | 92,977 | 90,884 | 108,371 | 178,168 | 77,266 | 547,666 |
| Farm 3 | 44,422 | 32,936 | 49,572 | 64,621 | 17,781 | 209,332 |
| Farm 4 | 451,824 | 156,580 | 193,701 | 726,947 | 191,333 | 1,720,385 |
| Total | 92,977 | 90,884 | 108,371 | 178,168 | 77,266 | 547,666 |

^a^Backup of the records during that period not available (NA)

**Table F:** **Estimated sigma and mean values with 95% CI for the Generalized Extreme Value Distribution of *R_0_* values for all control measures.**

| **Control #** | **k** | **95%CI (k)** | **sigma** | **95% (sigma)** | **Mean** | **95% CI** |
| --- | --- | --- | --- | --- | --- | --- |
| 0 | 0.5371 | 0.5272 - 0.5471 | 1.6199 | 1.6037-1.6364 | 1.7623 | 1.7456 - 1.7790 |
| 1 | 0.5356 | 0.5256 - 0.5456 | 1.6206 | 1.6043-1.6371 | 1.7557 | 1.7390 - 1.7725 |
| 2 | 0.5262 | 0.5163 - 0.5362 | 1.6100 | 1.5940-1.6262 | 1.7401 | 1.7235 - 1.7567 |
| 3 | 0.1332 | 0.1245 - 0.1419 | 0.6692 | 0.6638-0.6746 | 1.0262 | 1.0193 - 1.0332 |
| 4a | 0.2403 | 0.2314 - 0.2492 | 0.9521 | 0.9441-0.9602 | 1.3013 | 1.2915 - 1.3111 |
| 4b | 0.0017 | -0.0069-0.0102 | 0.5908 | 0.5862-0.5954 | 0.9685 | 0.9624 - 0.9746 |
| 5 | 0.5041 | 0.4945 - 0.5137 | 1.6006 | 1.5849-1.6164 | 1.7291 | 1.7127 - 1.7455 |
| 12 | 0.5236 | 0.5137 - 0.5335 | 1.6110 | 1.5950-1.6273 | 1.7378 | 1.7212 - 1.7543 |
| 13 | 0.1355 | 0.1267 -0.1444 | 0.6717 | 0.6662-0.6772 | 1.0120 | 1.0050 - 1.0189 |
| 14a | 0.2385 | 0.2296 –0.2475 | 0.9541 | 0.9460-0.9622 | 1.2948 | 1.2850 - 1.3047 |
| 14b | 0.0040 | -0.0047-0.1265 | 0.5923 | 0.5877-0.5969 | 0.9549 | 0.9488 - 0.9611 |
| 15 | 0.5037 | 0.4941 - 0.5133 | 1.5987 | 1.5831-1.6145 | 1.7269 | 1.7105 - 1.7432 |
| 23 | 0.1173 | 0.1085–0.1262 | 0.6911 | 0.6855-0.6967 | 1.0054 | 0.9982 - 1.0125 |
| 24a | 0.2275 | 0.2186-0.2364 | 0.9490 | 0.9410-0.9570 | 1.2740 | 1.2642 - 1.2838 |
| 24b | -0.0134 | -0.0222-0.0047 | 0.6076 | 0.6029-0.6124 | 0.9418 | 0.9355 - 0.9481 |
| 25 | 0.5041 | 0.4945-0.5137 | 1.6006 | 1.5849-1.6164 | 1.7291 | 1.7127 - 1.7455 |
| 34a | -0.0736 | -0.0823-(-0.0647) | 0.4878 | 0.4840-0.4915 | 0.8379 | 0.8328 - 0.8429 |
| 34b | -0.0113 | -0.0201-(-0.0026) | 0.5441 | 0.5398-0.5483 | 0.8990 | 0.8934 - 0.9047 |
| 35 | 0.2043 | 0.1956-0.2129 | 0.9436 | 0.9358-0.9514 | 1.2574 | 1.2477 - 1.2670 |
| 54a | 0.2043 | 0.1956-0.2129 | 0.9436 | 0.9358-0.9514 | 1.2574 | 1.2477 - 1.2670 |
| 54b | -0.0404 | -0.0488-(-0.0319) | 0.6248 | 0.6200-0.6296 | 0.9304 | 0.9240 - 0.9369 |
| 4a4b | -0.1278 | -0.1364-(-0.1193) | 0.4514 | 0.4480-0.4549 | 0.7997 | 0.7950 - 0.8043 |
| 1000 | -0.1913 | -0.2003-(-0.1824) | 0.4701 | 0.4664-0.4737 | 0.6972 | 0.6924 - 0.7021 |
| 24a4b | -0.1415 | -0.1507-(-0.1325) | 0.4626 | 0.4590-0.4663 | 0.7547 | 0.7499 - 0.7595 |
| 34a4b | -0.1341 | -0.1429-(-0.1252) | 0.4213 | 0.4180-0.4245 | 0.7408 | 0.7365 - 0.7452 |
| 54a4b | -0.1799 | -0.1887-(-0.1712) | 0.4838 | 0.4801-0.4876 | 0.7413 | 0.7363 - 0.7463 |
| 134a4b | -0.1221 | -0.1313-(-0.1129) | 0.4221 | 0.4188-0.4254 | 0.7242 | 0.7198 - 0.7286 |

Control measure 0 = No control measure ; control measure 1 = Colostrum management feeding colostrum replacer (CR) vs. maternal colostrum (MC); 2 = Offsite heifer-rearing; 3 = Reducing MAP bioburden in the environment by10-fold by scraping fecal slurry on hard surfaces or power washing; 4 = test and cull, scenario a: testing at dry off on a weekly basis and culling test-positive cows; scenario b = testing all the adult cows (lactating and dry) annually; 5 = Delaying exposure to infected cows at adult hood
